# [Au^III^(N^N)Br_2_](PF_6_): A Class of Antibacterial and Antibiofilm Complexes (N^N = 2,2′-Bipyridine and 1,10-Phenanthroline Derivatives)

**DOI:** 10.1021/acs.inorgchem.2c04410

**Published:** 2023-02-02

**Authors:** M. Carla Aragoni, Enrico Podda, Veronica Caria, Silvia A. Carta, M. Francesca Cherchi, Vito Lippolis, Simone Murgia, Germano Orrù, Gabriele Pippia, Alessandra Scano, Alexandra M. Z. Slawin, J. Derek Woollins, Anna Pintus, Massimiliano Arca

**Affiliations:** † Dipartimento di Scienze Chimiche e Geologiche, Università degli Studi di Cagliari, S. S. 554 bivio per Sestu, Monserrato Cagliari09042, Italy; ‡ Centro Servizi di Ateneo per la Ricerca (CeSAR), Università degli Studi di Cagliari, S. S. 554 bivio per Sestu, Monserrato Cagliari09042, Italy; § Dipartimento di Scienze Chirurgiche, University of Cagliari, Cagliari09042, Italy; ∥ EaStCHEM School of Chemistry, University of St. Andrews, North Haugh, Fife, St. AndrewsKY16 9ST, U.K.; ⊥ Department of Chemistry, Khalifa University, Abu Dhabi127788, United Arab Emirates

## Abstract

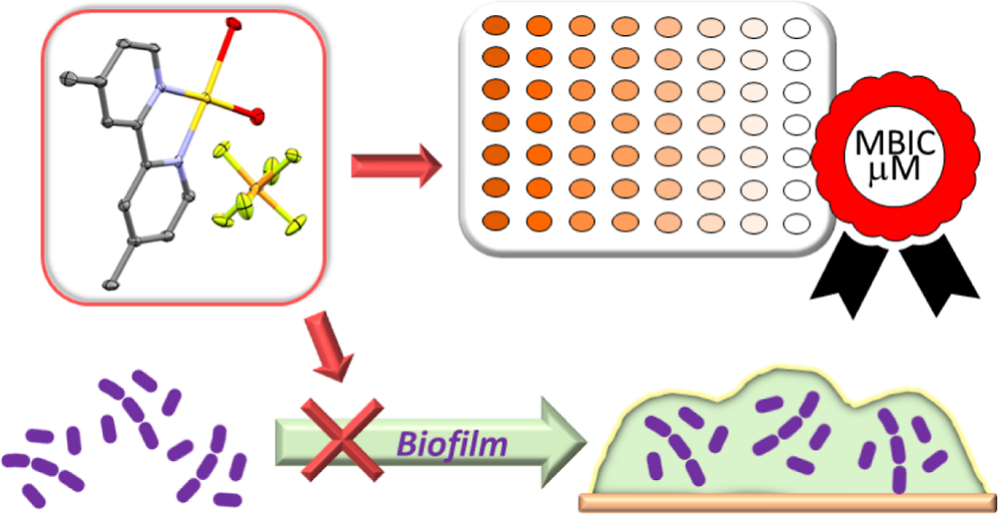

A series of new complexes of general formula [Au^III^(N^N)Br_2_](PF_6_) (N^N = 2,2′-bipyridine and 1,10-phenanthroline derivatives) were prepared and characterized by spectroscopic, electrochemical, and diffractometric techniques and tested against Gram-positive and Gram-negative bacterial strains (*Staphylococcus aureus*, *Streptococcus intermedius*, *Pseudomonas aeruginosa*, and *Escherichia coli*), showing promising antibacterial and antibiofilm properties.

## Abstract 2

[Au^III^(N^N)Br_2_](PF_6_) compounds (N^N = 2,2′-bipyridine and 1,10-phenanthroline derivatives) show wider antimicrobial and antibiofilm activities as compared to their chlorido analogues, particularly against anaerobic bacterial strains, and a redox mechanism of action along with an easier exchange of the bromido ligands can be hypothesized based on structure−property relationships, supported by DFT calculations.

## Introduction

Among their many uses, ranging from jewelry to catalysis, electronics, and nanotechnology,^1^ gold and its compounds have also been employed for medical applications since ancient times.^2,3^ Modern chrysotherapy^4^ is considered to have started in the middle of the 20^th^ century with the use of gold(I) complexes such as auranofin, solganol, and myochrisine as antiarthritic agents.^5^ Since then, gold compounds have been evaluated as therapeutical agents^6,7^ for the treatment of bronchial asthma,^7^ HIV,^8^ malaria,^9,10^ SARS-CoV-2,^11^ and cancer.^12−16^ The cytotoxic activity of both gold(I)^17^ and gold(III) complexes has been investigated, also prompted by the structural and electronic similarity of Au^III^ complexes with the largely tapped Pt^II^ analogues, although isoelectronic complexes containing the two metal ions show a different mechanism of action.^18,19^ Messori, Casini, and other authors demonstrated that the vast majority of cytotoxic Au^III^ complexes have a weaker affinity for DNA than Pt^II^ derivatives^20−22^ but are capable of interacting with several proteins,^21,22^ such as mitochondrial enzyme thioredoxin reductase,^23^ cysteine proteases,^24^ and human glutathione reductase.^25^

**Scheme 1:**
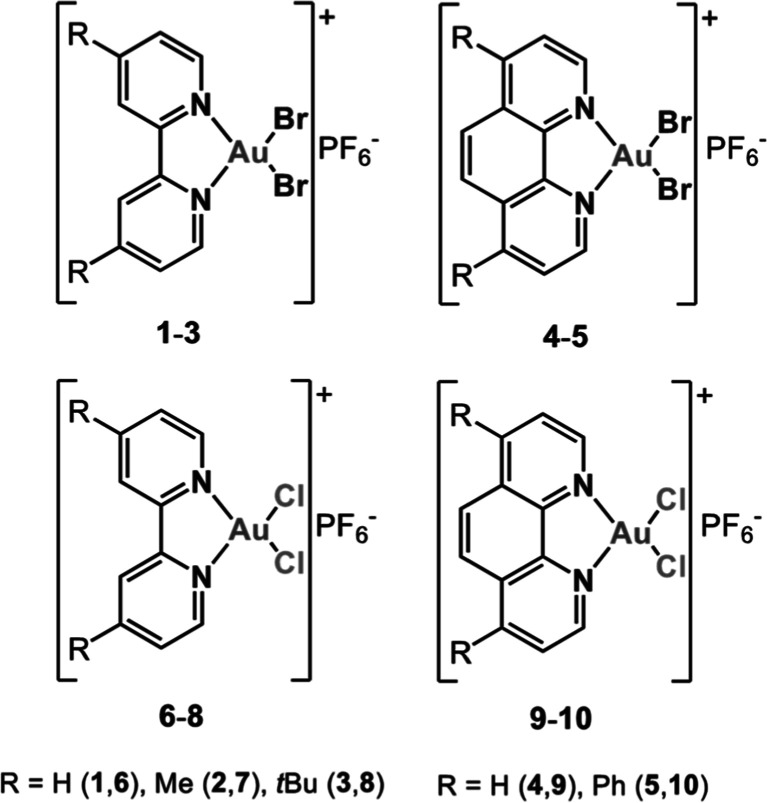
Molecular Structures of Compounds **1**–**10**

Reports on the antimicrobial activity of gold complexes are much fewer,^26^ even though metal-based compounds represent a very promising chemical scaffold in this field,^27^ since they can act against nonclassical targets and multiple bacterial sites simultaneously.^28,29^ This can prevent the acquisition of antimicrobial resistance,^30^ a serious threat and a huge financial burden to public health systems^31^ that is often worsened when bacteria are organized in complex sessile communities (biofilm).^32^ Several studies have been carried out on antimicrobic gold(I) compounds,^26^ showing in some cases promising results, especially against Gram-positive bacteria.^33,34^ The lesser number of reports on antimicrobial gold(III) complexes^34,35^ can be partly attributed to their tendency toward reduction and poor stability under physiological conditions.^36^ It is well known that chelating N^N and C^N ligands can effectively stabilize Au^III^ toward reduction.^37,38^ In this context, some of the authors recently reported on the antimicrobial activity against the *Staphylococcus* species of [Au(Py^b^-H)(mnt)] (Py^b^-H = C-deprotonated 2-benzylpyridine, mnt^2–^ = 1,2-dicyanoethene-1,2-dithiolate),^39^ a cycloaurated gold(III) complex showing antibiofilm properties. Following these previous studies, we report here on a series of gold(III)-chelated coordination compounds of general formula [Au(N^N)Br_2_](PF_6_), featuring 2,2′-bipyridine and 1,10-phenanthroline derivatives in combination with bromide ancillary ligands (Scheme 1), as promising antibacterial and antibiofilm compounds.

## Results and Discussion

### Synthesis

Compounds **1**–**5** were prepared by reacting the relevant N^N derivative with potassium tetrabromoaurate(III), generated in situ through the addition of KBr to a solution of KAuCl_4_,^40^ in the presence of an excess of potassium hexafluorophosphate. The already reported chlorido-analogues **6**–**10** have also been re-prepared for the sake of comparison.^41−45^

### Spectroscopic Characterization

The microanalytical and spectroscopic characterization of the products confirmed the general composition [Au(N^N)Br_2_](PF_6_). The peaks of the hexafluorophosphate anion^46^ are clearly visible in FT-IR spectra at about 840 and 557 cm^–1^ (Figure S1 for **3**). An upfield shift of about 0.2 ppm for the doublet corresponding to the protons in ortho-position with respect to the nitrogen atoms could be observed in the ^1^H NMR spectra on passing from compounds **1**–**5** to **6**–**10** (Figure S2 for **3** and **8**). UV–visible spectra recorded in MeCN solution for compounds **1**–**5** (Figure S3 for **1**) show intense absorption bands in the range 280–315 nm, slightly blue-shifted with respect to the corresponding ones of compounds **6**–**10** (300–330 nm), which can be assigned to ligand-to-metal charge-transfer transitions peculiar to the mononuclear square-planar gold(III) chromophore.^47^ The UV absorptions at about 220 nm can be interpreted as intra-ligand (IL) π–π* transitions.

### X-ray Diffraction Studies

**Figure 1:**
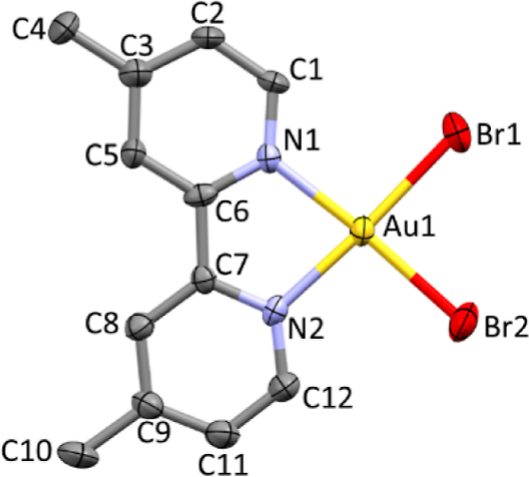
Drawing and atom labeling scheme for the complex cation in compound **2**. Thermal ellipsoids were shown at the 60% probability level. Hydrogen atoms were omitted for clarity.

The crystal structures of compounds **1**, **2**, **4**, and **5**·CH_2_Cl_2_ (Table S1), as well as those obtained for compounds **6** and **9** (confirming the previously reported crystallographic data),^48,49^ show the central gold(III) ion lying in a slightly distorted square-planar coordination environment. The complex cations [Au(N^N)Br_2_]^+^ are almost planar, with the exception of the phenyl substituents at the 1,10-phenanthroline ligand in **5**·CH_2_Cl_2_ (Figures 1, S4, and S5). The average Au–Cl (2.265 and 2.254 Å for **6** and **9**, respectively) and Au–Br distances (2.383, 2.377, 2.383, and 2.377 Å for **1**, **2**, **4**, and **5**·CH_2_Cl_2_, respectively) are similar to those reported for analogous [Au(N^N)X_2_]^+^ complexes (Tables S2 and S3)^42,48,50−55^ and show the expected increase of *d*_Au–X_ on passing from X = Cl to X = Br. In all structures, the packing is mainly governed by contacts between complex cations and hexafluorophosphate anions (Figure S6 for **4**). In the case of isostructural compounds **4** and **9**, a Au^III^···Au^III^ distance larger than the sum of van der Waals (vdW) radii but lower than Allingers’ radii^56^ could be envisaged (Au1···Au1′ = 3.642 and 3.547 Å for **4** and **9**, respectively; ′ = 1 – *x*, 1 – *y*, 2 – *z*), indicating a weak aurophilic interaction^50^ between couples of symmetry-related complex cations arranged in a head-to-tail fashion (Figure S7). Pairs of molecules engaging in aurophilic contacts interact with each other through weak slipped π–π interactions involving the phenanthroline rings [shortest ring-centroid distance for **4**: centroid···C11″, 3.65 Å; for **9**: centroid···C7″, 3.66 Å; ″ = −1/2 + *x*, *y*, 3/2 – *z*]. Compound **1** shows additional Br···Br contacts between couples of symmetry-related complex units, featuring a *d*_Br···Br_ = 3.657 Å close to the sum of vdW radii (Figure S8).

### Microbiological Tests

Antimicrobial tests against Gram-positive (*Staphylococcus aureus* and *Streptococcus intermedius*) and Gram-negative (*Pseudomonas aeruginosa* and *Escherichia coli*) bacteria were carried out on compounds **1**–**4** and **6**–**9** and the corresponding N^N free ligands (Table 1), while compounds **5** and **10** were not assayed for solubility reasons. It is worth mentioning that a systematic study on the antibacterial activity of these systems has never been carried out, although some studies were conducted on the biological activity of compounds **6**–**10**,^42,45,48,56−59^ and the antimicrobial properties of related [Au(N^N)Cl_2_]^+^ systems with different diimine ligands and counteranions were occasionally investigated.^34,60−62^

**Table 1:** Antibacterial MIC, MBC, and MBIC for Compounds **1**–**4** and **6**–**9** and the Diimine Ligands in mM Concentration and mg·mL^–1^ (in Parentheses)

	MIC				MBC				MBIC			
	*E. coli*	*S. aureus*	*P. aerug.*	*S. interm.*	*E. coli*	*S. aureus*	*P. aerug.*	*S. interm.*	*E. coli*	*S. aureus*	*P. aerug.*	*S. interm.*
**1**	5.0 (3.3)	>5.0 (>3.3)	5.0 (3.3)	>5.0 (>3.3)	>5.0 (>3.3)	>5.0 (>3.3)	5.0 (3.3)	>5.0 (>3.3)	5.0·10^–3^ (3.3·10^–3^)	5.0 (3.3)	0.050 (0.03)	5.0·10^–3^ (3.3·10^–3^)
**2**	5.0 (3.4)	>5.0 (>3.4)	5.0 (3.4)	0.50 (0.34)	5.0 (3.4)	>5.0 (>3.4)	5.0 (3.4)	5.0 (3.4)	5.0·10^–3^ (3.4·10^–3^)	>5.0 (>3.4)	5.0 (3.4)	5.0 (3.4)
**3**	5.0 (3.8)	>5.0 (>3.8)	>5.0 (>3.8)	>5.0 (>3.8)	>5.0 (>3.8)	>5.0 (>3.8)	>5.0 (>3.8)	>5.0 (>3.8)	0.050 (0.038)	>5.0 (>3.8)	0.50 (0.38)	5.0 (3.8)
**4**	0.50 (0.34)	0.50 (0.34)	0.50 (0.34)	0.50 (0.34)	0.50 (0.34)	0.50 (0.34)	0.50 (0.34)	0.50 (0.34)	0.50 (0.34)	0.50 (0.34)	0.50 (0.34)	0.50 (0.34)
**6**	0.50 (0.28)	0.50 (0.28)	0.50 (0.28)	>5.0 (>2.8)	0.50 (0.28)	5.0 (2.8)	5.0 (2.8)	>5.0 (>2.8)	0.50 (0.28)	0.50 (0.28)	>5.0 (>2.8)	5.0 (2.8)
**7**	0.50 (0.30)	>5.0 (>3.0)	0.50 (0.30)	5.0 (3.0)	>5.0 (>3.0)	>5.0 (>3.0)	5.0 (3.0)	>5.0 (>3.0)	0.50 (0.30)	>5.0 (>3.0)	0.50 (0.30)	>5.0 (>3.0)
**8**	>5.0 (>3.4)	>5.0 (>3.4)	>5.0 (>3.4)	>5.0 (>3.4)	>5.0 (>3.4)	>5.0 (>3.4)	>5.0 (>3.4)	>5.0 (>3.4)	>5.0 (>3.4)	>5.0 (>3.4)	>5.0 (>3.4)	>5.0 (>3.4)
**9**	0.50 (0.30)	0.50 (0.30)	>5.0 (>3.0)	5.0 (3.0)	0.50 (0.30)	5.0 (3.0)	>5.0 (>3.0)	>5.0 (>3.0)	0.50 (0.30)	0.50 (0.30)	>5.0 (>3.0)	0.50 (0.30)
bipy	5.0 (0.78)	5.0 (0.78)	5.0 (0.78)	>5.0 (>0.78)	>5.0 (>0.78)	>5.0 (>0.78)	>5.0 (>0.78)	>5.0 (>0.78)	5.0·10^–3^(7.8·10^–4^)	>5.0 (>0.78)	>5.0 (>0.78)	>5.0 (>0.78)
Me_2_bipy	>5.0 (>0.92)	>5.0 (>0.92)	>5.0 (>0.92)	>5.0 (>0.92)	>5.0 (>0.92)	>5.0 (>0.92)	>5.0 (>0.92)	>5.0 (>0.92)	>5.0 (>0.92)	0.50 (0.092)	>5.0 (>0.92)	0.50 (0.092)
*t*Bu_2_bipy	>5.0 (>1.34)	>5.0 (>1.34)	>5.0 (>1.34)	>5.0 (>1.34)	>5.0 (>1.34)	>5.0 (>1.34)	>5.0 (>1.34)	>5.0 (>1.34)	0.050 (0.013)	5.0 (1.3)	0.50 (0.13)	5.0 (1.3)
phen	5.0 (0.90)	5.0 (0.90)	5.0 (0.90)	>5.0 (>0.90)	>5.0 (>0.90)	5.0 (0.90)	5.0 (0.90)	>5.0 (>0.90)	0.050(9.0·10^–3^)	0.50 (0.090)	0.050(9.0·10^–3^)	>5.0 (>0.90)

The free ligands either did not display any growth-inhibiting/bactericidal properties or showed minimum inhibitory concentration (MIC) and minimum bactericidal concentration (MBC) values of about 5.0 mM. On the other hand, some of the complexes were found to be moderately active against the tested bacterial strains. Although no MIC and MBC values below 0.50 mM were observed, the experimental findings allow for some tentative rationalizations on quantitative structure–activity relationships (QSARs). Compounds **3** and **8** showed MICs ≥ 5.0 mM against all bacterial strains. This could be attributed to the presence of the bulky and hydrophobic *tert*-butyl substituents at the diimine ligand, which might reduce permeation into the bacterial cell and thus hamper the growth-inhibitory activity. Compound **7** was active only against the two Gram-negative strains, with MICs amounting to 0.50 mM (0.28–0.34 mg·mL^–1^), while compounds **4**, **6**, and **9** also showed the ability to inhibit the growth of Gram-positive *S. aureus*. On the other hand, compounds **2** and **4** were the only ones active against the other Gram-positive species, namely *S. intermedius*, thus suggesting that the introduction of bromide in the place of chloride anions as ancillary ligands might exert a role. Compound **4** is particularly promising, having inhibitory properties against all four bacterial strains tested, as confirmed by the MBC values (about 0.50 mM). Compound **4** was in fact the only complex showing bactericidal abilities below 5.0 mM, except for compounds **6** and **9** against *E. coli*. Whatever the mechanism of action of compound **4**, this suggests that it should be at least in part independent of the cell permeability and metabolism type of the targeted bacteria.

**Figure 2:**
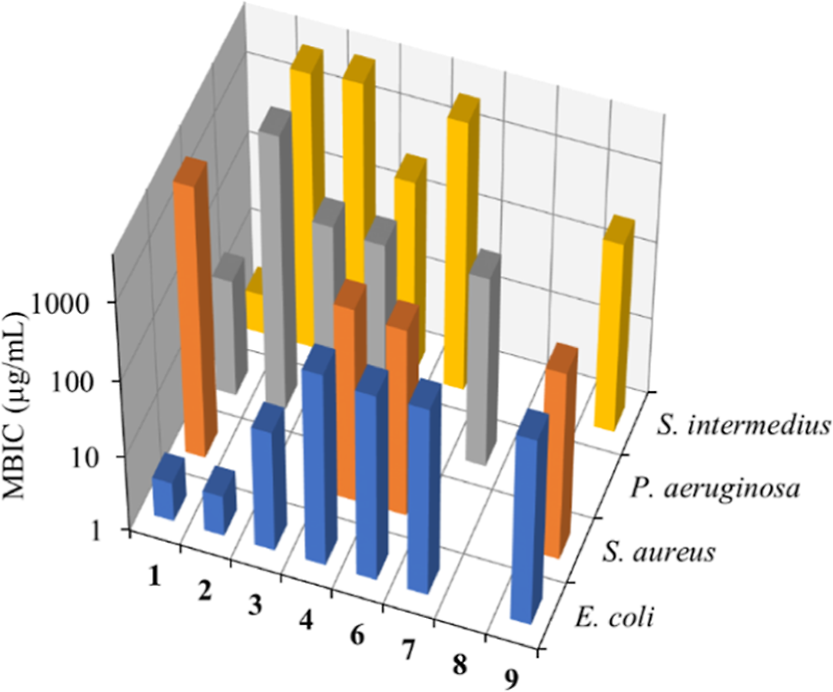
MBIC (μg·mL^–1^) for compounds **1**–**4** and **6**–**9** against different bacterial strains.

The most promising results were obtained in the antibiofilm tests, with most compounds showing the ability to inhibit the growth of biofilm in at least some of the investigated bacterial strains (Figure 2). Particularly low minimum biofilm inhibitory concentration (MBIC) values were observed in the case of **1**–**3** against *E. coli* (MBIC = 5.0–50 μM, 3.3–38 μg·mL^–1^), and compound **1** was also active against *P. aeruginosa* (MBIC = 50 μM, 30 μg·mL^–1^) and *S. intermedius* (MBIC = 5.0 μM, 3.3 μg·mL^–1^). These results suggest that the bromido complexes are the most promising candidates as antibiofilm agents.

### Electrochemistry

**Figure 3:**
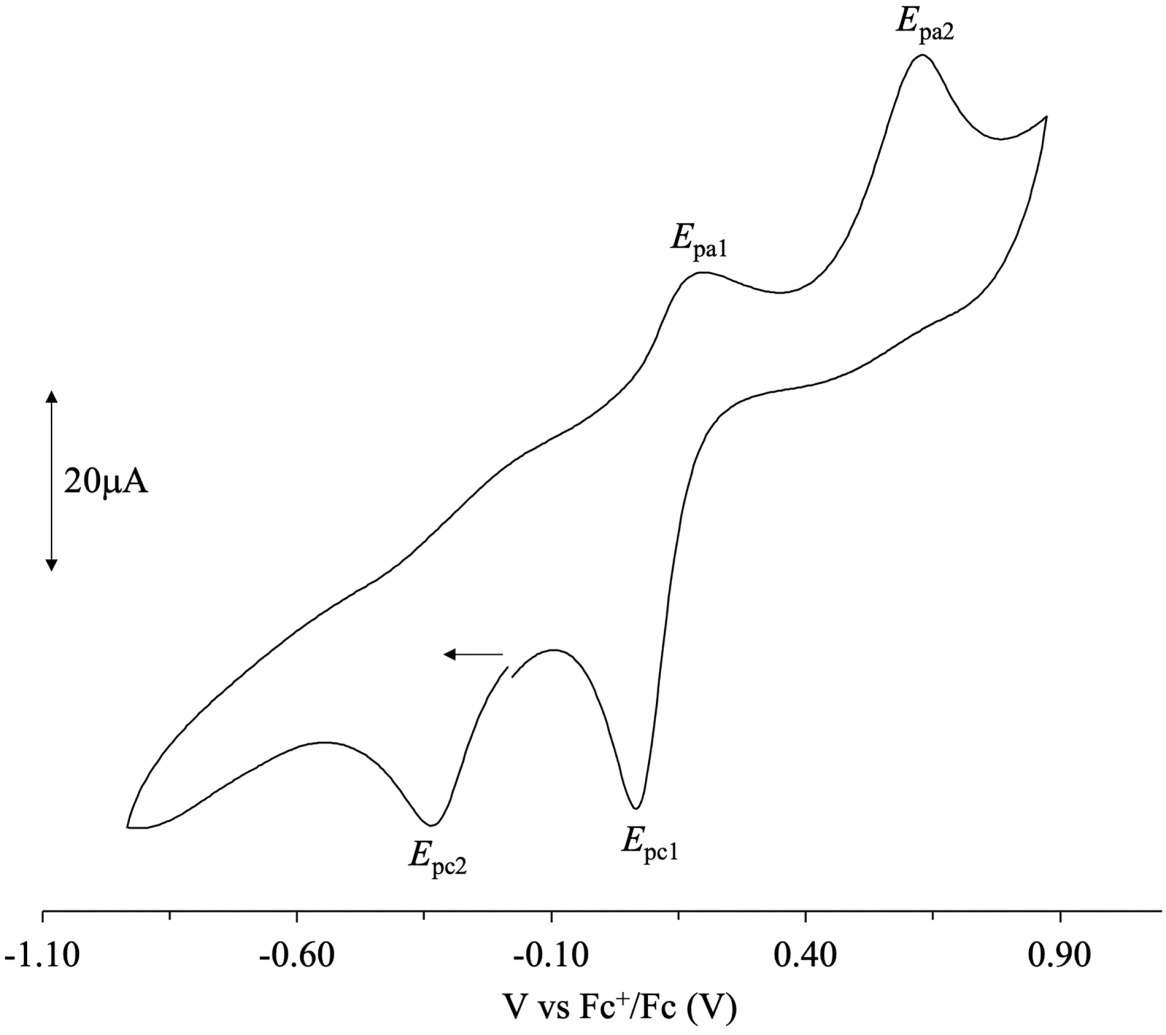
Cyclic voltammogram recorded for compound **6** (298 K; range, −0.90 to +0.90 V vs Fc^+^/Fc; supporting electrolyte, Bu_4_NPF_6_ 0.10 M). Under these conditions, no gold deposition was observed.

It has been pointed out that the biological activity of gold(III) complexes can be attributed to gold(I) metabolites generated by reduction of gold(III) compounds.^62,63^ In this context, reduction potentials are fundamental both in evaluating the stability of gold(III) species and in investigating the in vivo mechanism of action of metallodrugs.^19,64^ In particular, Casini et al. demonstrated that gold(III) dichlorido–diimine complexes, and compound **7** in particular, are active toward the A2780 human ovarian carcinoma cell line and the cisplatin–resistant variant A2780cisR, their mechanism of action being related to their reduction by amino acids.^42^ The electrochemical properties of compounds **1**–**4** and **6**–**9** were investigated by cyclic voltammetry (CV) measurements immediately after dissolution in MeCN solution, by adopting tetrabutylammonium hexafluorophosphate (0.1 M) as the supporting electrolyte (see Table 2 and Figure 3 for compound **6**). All potentials were referenced to the Fc^+^/Fc reversible redox couple.^65,66^

**Table 2:** KS-LUMO (ε_LUMO_, eV) and KS-HOMO (ε_HOMO_, eV) Eigenvalues Calculated in the Gas Phase at the DFT Level and Experimental CV Potentials *E* (vs Fc^+^/Fc) Recorded at a Platinum Electrode for Compounds **1**–**4** and **6**–**9** in MeCN Solution (Supporting Electrolyte Bu_4_NPF_6_ 0.1 M; 298 K; Scan Rate, 0.10 V·s^–1^)

	ε_HOMO_	ε_LUMO_	*E*_pc_1__	*E*_pc_2__	*E*_pc_3__^a^	*E*_pa_1__	*E*_pa_2__
**1**	–10.860	–7.1925	0.126	–0.387	–1.162		0.519
**2**	–10.645	–6.9356	0.083	–0.371	–1.113		0.390
**3**	–10.496	–6.7686	–0.013	–0.417	–0.899	0.197	
**4**	–10.826	–7.1310	0.094	–0.355	–0.975	0.158	0.358
**6**	–11.437	–7.1471	0.068	–0.328	–1.197	0.195	0.701
**7**	–11.204	–6.8763	–0.005	–0.386	–0.918		0.631
**8**	–11.045	–6.6943	0.012	–0.417	–0.977	0.188	
**9**	–11.368	–7.0891	0.054	–0.436	–0.967	0.147	0.590

a Reduction accompanied by gold deposition at the Pt electrode.

The cyclic voltammograms (scan rate = 0.10 V·s^–1^) show three clear irreversible cathodic peaks at average potentials *E*_pc_1__ = 0.05, *E*_pc_2__ = −0.39, and *E*_pc_3__ = −1.02 V versus Fc^+^/Fc, respectively, attributed to the Au^III^/Au^II^, Au^II^/Au^I^, and Au^I^/Au^0^ monoelectronic irreversible reduction steps, respectively (Table 2). The cathodic step at the lowest potential (*E*_pc_3__) was systematically accompanied by the deposition of a thin layer of metallic gold at the platinum electrode. The Au^I^/Au^0^ reduction, with gold deposition, was found in CH_2_Cl_2_ solution with similar values in a series of gold(III) complexes featuring the Hpbi ligand [−0.89, −0.92, −0.87, −0.85, and −0.90 V vs Fc^+^/Fc for [Au(pbi)Cl_2_], [Au(pbi)(AcO)_2_], [Au(pbi)Cl_2_]·AuCl, ([Au(pbi)Cl_2_]·AuPPh_3_)(PF_6_), and [Au(pbi)(AcO)_2_]·AuPPh_3_, respectively; Hpbi = 2-(2′-pyridyl)benzimidazole].^67,68^ Two successive reduction steps leading from gold(III) to gold(I) were reported for the two series of gold(III) dichlorido-diimine complexes [Au{(*S*,*S*)-R_2_eddip}Cl_2_](PF_6_) and [Au{(*S*,*S*)-R_2_eddch}Cl_2_](PF_6_) [(*S*,*S*)-R_2_eddip = (*S*,*S*)-ethylenediamine-*N*,*N*′-di-2-propanoate; (*S*,*S*)-R_2_eddch = (*S*,*S*)-ethylenediamine-*N*,*N*′-di-2(3-cyclohexyl)propanoate; R = Me, Et, n-Pr, and different saturated substituents], at average potentials of +0.13 and −0.56 V versus Ag/AgCl and +0.20 and −0.59 V versus Ag/AgCl for the former and latter series, respectively, in DMSO solution.^19^ The former cathodic process was attributed by the authors to the one-electron reduction to a short-lived Au^II^ intermediate formed by loss of a chloride ligand.^19^ Under anodic scan, most compounds displayed a peak at about *E*_pa_1__ = 0.20 V versus Fc^+^/Fc that can be tentatively attributed to the Au^II^/Au^III^ oxidation. A second irreversible anodic peak, independent of the reduction steps, could be observed in the range *E*_pa_2__ = 0.4 – 0.6 V versus Fc^+^/Fc for most compounds (Table 2).

A comparative examination of the CV results shows that all compounds are easily reduced, with *E*_pc_1__ mean values of 0.05 V versus Fc^+^/Fc. In general, chlorido ligands induce a very slight stabilization toward reduction as compared to the corresponding bromido complexes. On the other hand, *E*_pa_2__ values fall at more positive potentials for the chlorido-complexes as compared to the bromido ones.

As far as the effects of substitution on the N^N ligands are regarded, alkyl substituents stabilize the corresponding complexes toward reduction, unsubstituted derivatives **1** and **4** being the most easily reduced. The potentials associated with reduction to gold(I) species clearly show that compounds **3**, **7**, and **8** are less prone to reduction.

### Theoretical Calculations

**Figure 4:**
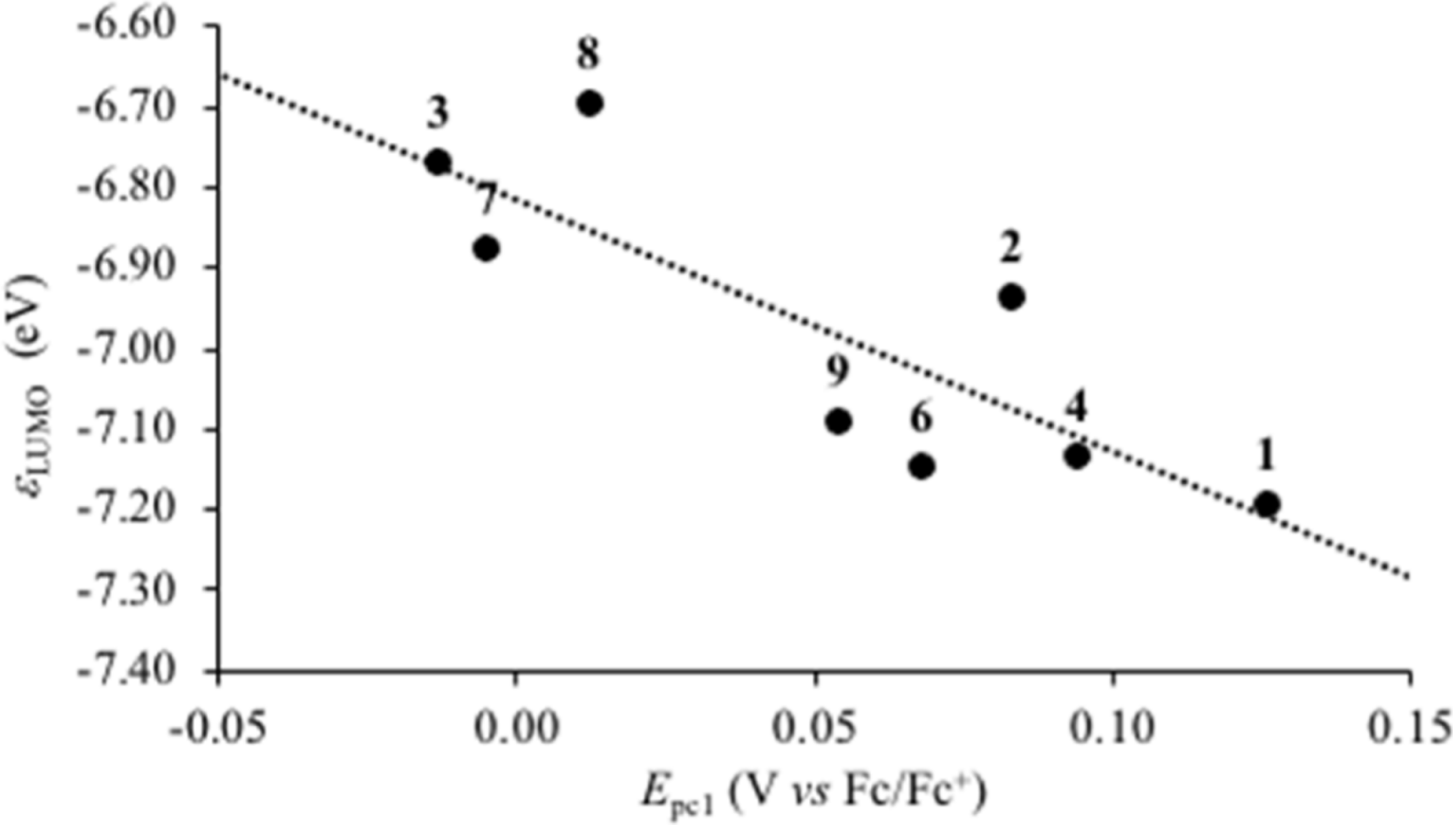
Correlation between *E*_pc_1__ reduction potentials determined by CV for compounds **1**–**4** and **6**–**9** and KS-LUMO eigenvalues ε_LUMO_ of their complex cations (*R*^2^ = 0.69).

Gold-based drugs can act as prodrugs that undergo ligand substitution or participate in redox reactions before interacting with their biotargets.^69−71^ Under physiological conditions, gold(III) complexes can be at least partly hydrolyzed to give their aqueous complexes.^69^ The mechanism of action of Au^III^ compounds is still a matter of debate and is under investigation.^69^ Some insights into the biological activity of the title compounds could be inferred from DFT calculations (Tables S4–S26 and Figures S9 and S10), carried out on the complex cations of compounds **1**–**10** based on previous studies on related systems.^39,72−75^ Analysis of the eigenvalues of Kohn–Sham (KS) frontier molecular orbitals at the optimized geometry (Table S25) shows that the complex cations of compounds **1**, **4**, **6**, and **9** feature the most stable lowest unoccupied molecular orbitals (KS-LUMOs), which are antibonding in nature with respect to the gold–halogen bonds (Figure S10). Calculated KS-LUMO eigenvalues (ε_LUMO_; Tables 2 and S25) can be related to the experimental reduction potentials *E*_pc_1__ (Figure 4), defining two groups of compounds: while the complex cations of compounds **3**, **7**, and **8** show the highest ε_LUMO_ values, resulting in less positive *E*_pc_1__ values, the remaining complexes feature lower ε_LUMO_ values, being therefore more prone to reduction. The tendency to reduction of these Au^III^ complexes to reduction might account for the activity of corresponding compounds **4**, **6**, and **9** against *S. aureus*, the only bacterial species among those tested featuring an oxidative metabolism, and more in general these results point at the systems with unsubstituted diimines as the most promising candidates against this bacterial species.

On the other hand, analysis of the highest-occupied KS molecular orbitals (KS-HOMOs; Tables 2 and S25) shows that HOMOs undergo a stabilization on passing from **1**–**5** to **6**–**10**. The less-negative eigenvalues of [Au(N^N)Br_2_]^+^ complex cations, reflected in less-positive *E*_pa_2__ values, may be related to the higher activity of bromido-complexes, in particular compounds **2** and **4**, against *S. intermedius* under anaerobic conditions, where reductive (fermentative) metabolism plays an important role and might also explain their higher antibiofilm capabilities (particularly **1**–**3**), since the inhibition of biofilm growth can also be associated with redox mechanisms.^76^ Recently, Re and co-workers evaluated theoretically the reactivity of gold(I) monocarbene complexes with protein targets in aqueous solution by considering the exchange reactions of neutral Au(I)NHC complexes with water and with the main binding sites in a protein or polypeptide.^77^ Analogously, in order to ascertain the influence of the halide on the reactivity of dihalido-diimine gold(III) complexes, the ease of replacement of halides by neutral or anionic interacting species X^*n*–^ (*n* = 0, 1) can be evaluated based on thermochemical data. The two following anion exchange equilibria can be considered
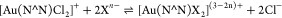
1
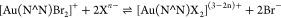
2

The spontaneity of each equilibrium can be estimated by the relevant free energy variations, Δ*G*_r,Cl_ and Δ*G*_r,Br_, respectively. Whatever the nature of X, the difference Δ*G*_r,Cl_ – Δ*G*_r,Br_ can be evaluated by considering free energy variation Δ*G*_Cl–Br_ = Δ*G*_r,Cl_ – Δ*G*_r,Br_ of the following halide exchange reaction
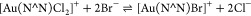
3

The analysis of thermochemical data for these reactions, calculated in aqueous media, shows that, independent of the nature of the diimine N^N, ΔG_Cl–Br_ is calculated to be positive by about 7 kcal·mol^–1^ (Table S26) in water solution, thus showing that bromide anions are more easily replaced than chlorides. This supports the hypothesis that, neglecting kinetic effects, [Au(N^N)Br_2_]^+^ complexes are more prone than the chlorido analogues to exchange reactions in aqueous solution with protein-binding sites.

## Conclusions

A class of dibromido-diimine gold(III) complexes of general formula [Au(N^N)Br_2_](PF_6_) were tested for their antibacterial properties against both Gram-positive and Gram-negative bacterial strains, showing very promising antibiofilm activities compared to their chlorido analogues, testified by MBIC values in the μM range. The different activities of the investigated library of complexes allowed for the formulation of QSARs also based on DFT calculations. Several factors may contribute to the antimicrobial properties of the new dibromid-diimineo complexes, including steric effects, tendency to anion exchange, and redox activity. A nice correlation holds between the reduction potentials *E*_pc_1__ and the KS-LUMO eigenvalues, showing that the variation in the electronic structure is responsible for the observed trend. Similar considerations were drawn for KS-HOMO eigenvalues and oxidation potentials, and these data can be reconciled with some of the trends observed for the antimicrobial activity. From the one side, the correlation between the electrochemical properties, the calculated frontier molecular orbital eigenvalues, and the antibacterial properties tentatively suggests a possible mechanism at least partly redox in nature. In addition, thermochemical data clarify the role of the halide, bromide ions being more prone to exchange reactions and therefore potentially more reactive toward active sites, such as amino acid residues in proteins. These preliminary rationalizations will pave the way to the preparation of yet more active compounds, and future work will also include the extension of these studies to additional bacteria.

## Experimental Part

### Materials and Methods

Solvents (reagent-grade) were purchased from Honeywell and used without further purification. Deuterated acetonitrile (CD_3_CN) was purchased from Eurisotop and stored under molecular sieves prior to use. Reagents were purchased from Honeywell, Alfa Aesar, Acros Organics, Chempur, Fluorochem, and Merck and used without further purification. Melting points are uncorrected and were carried out in capillaries on Electrothermal (up to 240 °C) and FALC mod. C (up to 290 °C) melting point apparatuses. Elemental analyses were performed with a PE 2400 series II CHNS/O elemental analyzer (*T* = 925 °C). FT-IR spectra were recorded with a Thermo-Nicolet 5700 spectrometer at room temperature. KBr pellets with a KBr beam splitter and KBr windows (4000–400 cm^–1^, resolution 4 cm^–1^) were used. UV–vis absorption spectra were recorded at 25 °C in a quartz cell of 10.00 mm optical path with a Thermo Evolution 300 (190–1100 nm) spectrophotometer. ^1^H NMR measurements were carried out in CD_3_CN at 25 °C, using a Bruker Avance 300 MHz (7.05 T) and Bruker Avance III HD 600 MHz (14.1 T) spectrometers operating at the operating frequencies of 300.13 and 600 MHz, respectively. Chemical shifts are reported in ppm (δ) and are calibrated to the solvent residue. Cyclic voltammetry experiments were recorded using a three-electrode cell, with a combined platinum working and counter-electrode and a standard Ag/AgCl (in KCl 3.5 M; 0.2223 V vs SHE at 25 °C) reference electrode. The experiments were performed at room temperature under an argon atmosphere in anhydrous MeCN with Bu_4_NPF_6_ (0.1 M) as the supporting electrolyte, at a potential scan rate of 0.10 V·s^–1^. Experiments were carried out on a Metrohm Autolab PGSTAT 10 potentiostat-galvanostat using model GPES electrochemical analysis software. All potential values are referenced to the bis-cyclopentadienyl-iron(III)/iron(II) couple (Fc^+^/Fc, *E*_1/2_ = +0.43 V vs Ag/AgCl under experimental conditions).^65,66^

### X-ray Diffraction Measurements

X-ray single-crystal diffraction data for compounds **6** and **9** were collected using a Rigaku Mercury70 CCD and a Rigaku XtaLAB P200 diffractometer operating at *T* = 93 and 173 K, respectively, and using Mo *K*α radiation. The data were indexed and processed using CrystalClear.^78^ The structure was solved with the ShelXS97^79^ solution program using direct methods and by using CrystalStructure 4.0 as the graphical interface.^80^ X-ray single-crystal diffraction data for compounds **1**, **2**, and **5**·CH_2_Cl_2_ were collected on a Bruker D8 Venture diffractometer equipped with a PHOTON II area detector operating at *T* = 100 K, for **1** and **2**, and at *T* = 298 K for **5**. The data were indexed and processed using Bruker SAINT^81^ and SADABS.^82^ The structures were solved with the ShelXT 2018^83^ solution program using dual-space methods and by using Olex2 1.5^84^ as the graphical interface. For compound **2**, all the screened crystals were twinned, and a satisfactory model was obtained by refining the data as a two-component twin. Moreover, the PF_6_^–^ anion in **2** is disordered and was modeled over two sites with fractional occupancies 79:21 using thermal and geometrical restraints. Similarly, compound **5** features a disordered PF_6_^–^ anion that was modeled over three sites with atomic occupancies 55:28:17. The dichloromethane molecule in **5**·CH_2_Cl_2_ is disordered, and the Cl atoms were modeled over two positions with atomic occupancies 59:41. X-ray single-crystal diffraction data for compound **4** were collected using a Rigaku XtaLAB P200 diffractometer operating at *T* = 173 K and using Mo *K*α radiation. The data were indexed and processed using CrystalClear v. 2.1.^78^ and REQAB.^85^ The structure was solved with the ShelXT 2018^83^ solution program using dual-space methods and by using CrystalStructure 4.3^80^ as the graphical interface. The models were refined with ShelXL 2018^86^ using full-matrix least-squares minimization on *F*^2^. All nonhydrogen atoms were refined anisotropically. Hydrogen atom positions were calculated geometrically and refined using the riding model. CCDC 2205798–2205803 contain the supplementary crystallographic data for this paper. These data can be obtained free of charge from the Cambridge Crystallographic Data Centre.

### Microbiological Assays

The following species were used: (i) Gram-positive bacteria, *S. aureus* ATCC 6538 (American Type Culture Collection), *S. intermedius* DSM 20573 (German Collection of Microorganism and cell culture); (ii) Gram-negative bacteria, *E. coli* ATCC 7075, and *P. aeruginosa* ATCC 27853. In vitro susceptibility testing was carried out using the MIC and MBC, which were determined in accordance with the European Committee for Antimicrobial Susceptibility Testing (EUCAST). The MIC and MBC procedures were performed using the microplate dilution technique. An inoculum of 10^6^ organisms/mL was applied, and the plates were examined for microbial growth after incubation for 48 h at 37 °C. For the biofilm evaluation, we used the protocol described by Montana University’s Center for Biofilm Engineering. A microplate containing serial concentrations of the compound, inoculated with the bacterial strains, was incubated at 37 °C for 6 days, to permit the biofilm formation. The plate samples were subsequently washed three times with phosphate-buffered saline GIBCO PBS (Thermo Fisher) to eliminate planktonic cells; the biofilm was stained with 100 μL of 0.1% w/v of crystal violet solution (Microbial, Uta, Italy) for 10 min at 25 °C; after three washes with PBS solution, 200 μL of 30% v/v acetic acid was added in every well to solubilize the dye from the bacterial biomass. The biofilm amount was measured with a plate reader spectrophotometer (SLT-Spectra II, SLT Instruments, Germany) at 620 nm.

### Computational Details

The computational investigation on the complex cations of **1**–**10** was carried out at the DFT level^87^ by adopting the Gaussian 16^88^ suite of programs. Following the results of previously reported calculations on related systems,^39,72−75^ the PBE0^89^ hybrid functional was adopted, along with the full-electron split valence basis sets (BSs) def2-SVP^90^ for light atomic species (C, H, N, Cl, and Br) and CRENBL basis sets^91^ with RECPs^92,93^ for heavier gold species. BS data were extracted from the EMSL BS Library.^94^ The molecular geometry optimizations (Tables S4–S23) were performed starting from structural data, when available, and were regularized by letting the model complexes belong to an ideal *C*_*2v*_ (**1**–**4**, **6**–**9**) or *C*_s_ (**5**, **10**) point group. Good agreement was found between the optimized (Table S24) and structural data (Tables S2 and S3), with only the Au–N bond distances being slightly overestimated (by less than 0.05 Å). Solvation calculations in water were also carried out at the same level of theory, by using the integral equation formalism of the polarizable continuous model (IEF-PCM) within the self-consistent reaction field (SCRF) approach,^95^ and a comparison between the structures optimized in the gas phase and in water showed negligible differences (Table S24). Harmonic frequency calculations were carried out to verify the nature of the minima of each optimized geometry. Thermochemical calculations (*T* = 298 K) were carried out to analyze the free energy variation related to eq 3. The programs GaussView 6.0.16^96^ and Chemissian 4.53^97^ were used to investigate the optimized structures and molecular orbital shapes.

### Synthesis

#### General Procedure for the Synthesis of Compounds **1**–**5**

KAuBr_4_ was generated in situ by adding a fourfold excess of KBr to an aqueous solution of KAuCl_4_.^40^ An equimolar solution of the desired diimine in CH_3_CN was then added, followed by an excess of KPF_6_. The resulting mixture was stirred for several hours at room temperature, and the resulting precipitate was collected by filtration, washed with water, toluene, and diethyl ether, and dried.

#### [Au(bipy)Br_2_](PF_6_) (**1**)

A solution of 2,2′-bipyridine (0.102 g, 0.654 mmol) in 5.0 mL of CH_3_CN was added to an aqueous solution (25 mL) of equimolar KAuBr_4_ and an excess of KPF_6_ (3.11 g, 16.9 mmol). Crystals suitable for X-ray diffraction were obtained by slow evaporation of a solution of the compound in CH_3_CN. Yield 0.398 g (93%). mp: 276 °C with decomposition. FT-IR: ν̃ = 3124 (vw), 3093 (w), 1064 (m), 1569 (w), 1506 (m), 1473 (m), 1454 (s), 1325 (m), 1317 (m), 1288 (w), 1274 (w), 1247 (m), 1207 (vw), 1181 (w), 1167 (m), 1132 (vw), 1114 (m), 1075 (m), 1045 (m), 1029 (m), 893 (m), 835 (vs), 767 (s), 713 (m), 674 (vw), 655 (w), 557 (s), 413 cm^–1^ (w). UV–vis–NIR (CH_3_CN): λ (ε) = 199 (30,000), 233 (54,000), 316 (11,700), 327 nm (11,100 M^–1^·cm^–1^). Elemental analysis calcd (%) for C_10_H_8_AuBr_2_F_6_N_2_P: C, 18.26; H, 1.23; N, 4.26. Found: C, 18.28; H, 1.18; N, 4.31. ^1^H NMR (600 MHz, CD_3_CN): δ = 9.69 (d, 2H), 8.58–8.56 (m, 4H), 8.02 (t, 2H) ppm.

#### [Au(Me_2_bipy)Br_2_](PF_6_) (**2**)

A solution of 4,4′-dimethyl-2,2′-bipyridine (0.110 g, 0.611 mmol) in 5.0 mL of CH_3_CN was added to an aqueous solution (25 mL) of equimolar KAuBr_4_ and an excess of KPF_6_ (3.50 g, 18.0 mmol). Crystals suitable for X-ray diffraction were obtained by slow evaporation of a solution of the compound in CH_3_CN. Yield 0.358 g (85%). mp: 206 °C with decomposition. FT-IR: ν̃ = 3132 (vw), 3080 (w), 1958 (vw), 1805 (vw), 1622 (s), 1564 (w), 1506 (w), 1489 (m), 1434 (m), 1376 (vw), 1324 (w), 1307 (w), 1290 (m), 1245 (w), 1221 (w), 1083 (w), 1048 (w), 1035 (m), 930 (w), 900 (m), 843 (vs), 741 (w), 714 (vw), 579 (m), 557 (s), 517 cm^–1^ (m). UV–vis–NIR (CH_3_CN): λ (ε) = 214 (37,200), 237 (55,000), 311 (11,500), 322 nm (11,000 M^–1^·cm^–1^). Elemental analysis calcd (%) for C_12_H_12_AuBr_2_F_6_N_2_P: C, 21.01; H, 1.76; N, 4.08. Found: C, 20.79; H, 1.54; N, 4.23. ^1^H NMR (600 MHz, CD_3_CN): δ = 9.47 (d, 2H), 8.37 (s, 2H), 7.80 (d, 2H), 2.67 (s, 6H) ppm.

#### [Au(*t*Bu_2_bipy)Br_2_](PF_6_) (**3**)

A solution of 4,4′-di-*tert*-butyl-2,2′-bipyridine (0.175 g, 0.652 mmol) in 5.0 mL of CH_3_CN was added to an aqueous solution (25 mL) of equimolar KAuBr_4_ and an excess of KPF_6_ (3.26 g, 17.1 mmol). Yield 0.452 g (90%). mp > 240 °C with decomposition. FT-IR: ν̃ = 2968 (m), 1617 (m), 1548 (vw), 1482 (w), 1421 (m), 1368 (w), 1254 (m), 1078 (w), 1047 (w), 1031 (w), 903 (w), 836 (vs), 597 (w), 557 cm^–1^ (s). UV–vis–NIR (CH_3_CN): λ (ε) = 204 (33,700), 240 (19,600), 279 (13,500), 319 nm (2400 M^–1^·cm^–1^). Elemental analysis calcd (%) for C_18_H_24_AuBr_2_F_6_N_2_P: C, 28.07; H, 3.14; N, 3.64. Found: C, 27.61; H, 3.26; N, 3.84. ^1^H NMR (300 MHz, CD_3_CN): δ = 9.53 (d, 2H), 8.50 (s, 2H), 7.97 (d, 2H), 1.50 (s, 18H) ppm.

#### [Au(phen)Br_2_](PF_6_) (**4**)

A solution of 1,10-phenanthroline (0.175 g, 0.973 mmol) in 5.0 mL of CH_3_CN was added to an aqueous solution (25 mL) of equimolar KAuBr_4_ and an excess of KPF_6_ (3.15 g, 17.1 mmol). Crystals suitable for X-ray diffraction were obtained by slow infusion of diethyl ether into a solution of the product in CH_3_CN. Yield 0.359 g (73%). mp: 239 °C with decomposition. FT-IR: ν̃ = 3093 (w), 1605 (w), 1585 (vw), 1522 (m), 1456 (vw), 1436 (m), 1449 (w), 1424 (w), 1221 (w), 1153 (w), 1114 (vw), 1102 (vw), 850 (vs), 838 (vs), 750 (vs), 703 (m), 557 cm^–1^ (s). UV–vis–NIR (CH_3_CN): λ (ε) = 207 (45,400), 223 (54,700), 281 nm (26,300 M^–1^·cm^–1^). Elemental analysis calcd (%) for C_12_H_8_AuBr_2_F_6_N_2_P: C, 21.14; H, 1.18; N, 4.11. Found: C, 20.99; H, 1.21; N, 4.05. ^1^H NMR (600 MHz, CD_3_CN): δ = 9.94 (s, 2H), 9.16 (d, 2H), 8.38 (s, 2H), 8.32 (t, 2H) ppm.

#### [Au(Ph_2_phen)Br_2_](PF_6_) (**5**)

A solution of 4,7-diphenyl-1,10-phenanthroline (0.213 g, 0.640 mmol) in 15 mL of CH_3_CN was added to an aqueous solution (95 mL) of equimolar KAuBr_4_ and an excess of KPF_6_ (3.10 g, 16.8 mmol). The crude product was recrystallized from CH_2_Cl_2_, thus obtaining crystals suitable for X-ray diffraction of species **5**·CH_2_Cl_2_. Yield 0.224 g (40%). mp: 247 °C with decomposition. FT-IR: ν̃ = 3080 (vw), 1624 (vw), 1598 (w), 1581 (vw), 1565 (m), 1519 (w), 1445 (vw), 1427 (m), 1403 (m), 1359 (w), 1262 (w), 1228 (w), 1093 (vw), 1018 (vw), 832 (vs), 763 (w), 728 (m), 670 (m), 669 (w), 640 (w), 559 cm^–1^ (s). UV–vis–NIR (CH_3_CN): λ (ε) = 235 (44,400), 300 nm (44,800 M^–1^·cm^–1^). Elemental analysis calcd (%) for C_24_H_16_AuBr_2_F_6_N_2_P: C, 34.56; H, 1.93; N, 3.36. Found: C, 33.98; H, 1.91; N, 3.36. ^1^H NMR (600 MHz, CD_3_CN): δ = 10.0 (d, 2H), 8.28 (m, 4H), 7.75 (m, 10H) ppm.

### General Procedure for the Synthesis of Compounds **6**–**10**

Compounds **6** and **9** were prepared as previously reported.^41,48,49^ Compounds **7**, **8**, and **10** were synthesized by adapting previously reported procedures.^41−45^

#### [Au(Me_2_bipy)Cl_2_](PF_6_) (**7**)

A solution of 4,4′-dimethyl-2,2′-bipyridine (0.122 g, 0.664 mmol) in 5.0 mL of CH_3_CN was added to an aqueous solution (25 mL) of equimolar KAuCl_4_ and an excess of KPF_6_ (3.26 g, 17.7 mmol). Yield 0.349 g (88%). mp: 200 °C with decomposition. FT-IR: ν̃ = 3088 (vw), 2925 (vw), 1621 (m), 1565 (vw), 1507 (vw), 1488 (vw), 1448 (w), 1383 (vw), 1325 (vw), 1307 (vw), 1291 (vw), 1247 (vw), 1221 (vw), 1084 (vw), 1056 (vw), 1039 (vw), 843 (vs), 558 (s), 518 cm^–1^ (w). UV–vis–NIR (CH_3_CN): λ (ε) = 208 (33,500), 227 (31,000), 308 nm (8000 M^–1^·cm^–1^). Elemental analysis calcd (%) for C_12_H_12_AuCl_2_F_6_N_2_P: C, 24.14; H, 2.03; N, 4.69. Found: C, 24.15; H, 1.92; N, 4.77. ^1^H NMR (300 MHz, CD_3_CN): δ = 9.29 (d, 2H), 8.45 (s, 2H), 7.90 (d, 2H), 2.74 (s, 6H) ppm.

#### [Au(*t*Bu_2_bipy)Cl_2_](PF_6_) (**8**)

A solution of 4,4′-di-*tert*-butyl-2,2′-bipyridine (0.177 g, 0.660 mmol) in 5.0 mL of CH_3_CN was added to an aqueous solution (25 mL) of equimolar KAuCl_4_ and an excess of KPF_6_ (3.04 g, 16.5 mmol). Yield 0.351 g (89%). mp > 240 °C with decomposition. FT-IR: ν̃ = 3093 (vw), 2970 (m), 1619 (m), 1553 (w), 1480 (w), 1424 (m), 1370 (w), 1301 (vw), 1255 (m), 1205 (vw), 1121 (vw), 1079 (w), 1053 (vw), 1027 (vw), 904 (vw), 836 (vs), 596 (w), 557 (s), 472 cm^–1^ (w). UV–vis–NIR (CH_3_CN): λ (ε) = 208 (33,500), 227 (31,000), 308 nm (8000 M^–1^·cm^–1^). Elemental analysis calcd (%) for C_18_H_24_AuCl_2_F_6_N_2_P: C, 31.74; H, 3.55; N, 4.11. Found: C, 30.98; H, 2.77; N, 4.08. ^1^H NMR (300 MHz, CD_3_CN): δ = 9.34 (d, 2H), 8.58 (s, 2H), 8.06 (d, 2H), 1.55 (s, 18H) ppm.

#### [Au(Ph_2_phen)Cl_2_](PF_6_) (**10**)

A solution of 4,7-diphenyl-1,10-phenanthroline (0.218 g, 0.654 mmol) in 5.0 mL of CH_3_CN was added to an aqueous solution (25 mL) of equimolar KAuCl_4_ and an excess of KPF_6_ (3.09 g, 16.8 mmol). Yield 0.459 g (94%). mp > 240 °C. FT-IR: ν̃ = 3677 (w), 3596 (w), 3089 (w), 1627 (vw), 1600 (m), 1581 (vw), 1567 (m), 1517 (m), 1496 (vw), 1446 (vw), 1428 (m), 1406 (m), 1361 (w), 1300 (vw), 1282 (vw), 1230 (m), 1192 (vw), 1019 (vw), 999 (vw), 834 (vs), 765 (m), 730 (w), 705 (m), 671 (w), 642 (w), 558 cm^–1^ (s). UV–vis–NIR (CH_3_CN): λ (ε) = 222 (64,000), 298 nm (33,400 M^–1^·cm^–1^). Elemental analysis calcd (%) for C_24_H_16_AuCl_2_F_6_N_2_P: C, 38.68; H, 2.16; N, 3.76. Found: C, 38.55; H, 2.03; N, 3.31. ^1^H NMR (300 MHz, CD_3_CN): δ = 9.73 (d, 2H), 8.29 (d, 2H), 8.27 (d, 2H), 7.73 (m, 10H) ppm.

## Supporting Information Available

Crystallographic data and packing details for **1**, **2**, **4**, **5**·CH_2_Cl_2_, **6**, and **9**; DFT-optimized geometries and metric parameters; eigenvalues and drawings of frontier molecular orbitals; calculated thermochemical data; representative FT-IR, UV-Vis, and ^1^H NMR spectra (PDF)
