# Supplementary material for: [AuIII(N^N)Br2](PF6): A Class of Antibacterial and Antibiofilm Complexes (N^N = 2,2′-Bipyridine and 1,10-Phenanthroline Derivatives)
Source: Inorg Chem. 2023 Feb 2;62(6):2924–33. doi: 10.1021/acs.inorgchem.2c04410 (PMC9930124; doi:10.1021/acs.inorgchem.2c04410)
Supplement: Supplementary file 1 — ic2c04410_si_001.pdf [file ic2c04410_si_001.pdf]

Supplementary material

$[Au^{III}(N^\wedge N)Br_2](PF_6)$: a class of antibacterial and antibiofilm complexes (N^N = 2,2'-bipyridine and 1,10-phenanthroline derivatives)

M. Carla Aragoni,[a] Enrico Podda,[a,b] Veronica Caria,[a] Silvia A. Carta,[a] M. Francesca Cherchi,[a] Vito Lippolis,[a] Simone Murgia,[a] Germano Orrù,[c] Gabriele Pippia,[a] Alessandra Scano,[c] Alexandra M. Z. Slawin,[d] J. Derek Woollins,[d,e] Anna Pintus,[a,*] and Massimiliano Arca[a,*]

[a] Dipartimento di Scienze Chimiche e Geologiche, Università degli Studi di Cagliari, S. S. 554 Bivio per Sestu, 09042 Monserrato (Cagliari), Italy

[b] Centro Servizi di Ateneo per la Ricerca (CeSAR), Università degli Studi di Cagliari, S. S. 554 bivio per Sestu, 09042 Monserrato (Cagliari), Italy

[c] Dipartimento di Scienze Chirurgiche, University of Cagliari, 09042 Cagliari, Italy

[d] EaStCHEM School of Chemistry, University of St. Andrews, North Haugh, St. Andrews, Fife, UK, KY16 9ST

[e] Department of Chemistry, Khalifa University, Abu Dhabi 127788, United Arab Emirates

[*] marca@unica.it, apintus@unica.it.

Table S1. Crystal data for **1**, **2**, **4**, **5**·CH_2Cl_2, **6**, and **9**.

	**1**	**2**	**4**	**5**·CH_2Cl_2	**6**	**9**
Empirical formula	$C_{10}H_8AuBr_2F_6N_2P$	$C_{12}H_{12}AuBr_2F_6N_2P$	$C_{12}H_8AuBr_2F_6N_2P$	$C_{25}H_{18}AuBr_2Cl_2F_6N_2P$	$C_{10}H_8AuCl_2F_6N_2P$	$C_{12}H_8AuCl_2F_6N_2P$
Calculated density (g·cm^{-3})	2.852	2.588	2.820	2.129	2.661	2.527
Formula Weight	657.94	685.99	681.96	919.12	569.02	593.04
Crystal size (mm)	0.19×0.15×0.10	0.10×0.10×0.03	0.10×0.10×0.08	0.60×0.18×0.16	0.10×0.03×0.03	0.15×0.09×0.02
T (K)	100(2)	100(2)	173(2)	298(2)	93(2)	173(2)
Crystal System	orthorhombic	monoclinic	orthorhombic	monoclinic	monoclinic	orthorhombic
Space Group	*Pbca*	$P2_1/c$	*Pbca*	$P2_1/c$	$P2_1/n$	*Pbca*
a (Å)	13.7594(7)	7.682(3)	13.0850(11)	17.0977(12)	6.6949(17)	12.9981(14)
b (Å)	13.6594(7)	11.872(5)	15.5551(13)	7.8822(5)	14.778(3)	15.4073(16)
c (Å)	16.3042(8)	19.660(10)	15.7809(13)	21.7184(15)	14.625(3)	15.5654(16)
α (°)	90	90	90	90	90	90
β (°)	90	100.972(14)	90	101.605(3)	101.048(6)	90
γ (°)	90	90	90	90	90	90
V (Å^3)	3064.3(3)	1760.3(14)	3212.0(5)	2867.1(3)	1420.1(6)	3117.2(6)
Z	8	4	8	4	4	8
Z'	1	1	1	1	1	1
Wavelength (Å)	0.71073	0.71073	0.71075	0.71073	0.71075	0.71075
Radiation type	Mo $K\alpha$	Mo $K\alpha$	Mo $K\alpha$	Mo $K\alpha$	Mo $K\alpha$	Mo $K\alpha$
Total number of data	54956	38486	14996	57341	9270	35753
Unique reflections	5580	4098	2916	6575	2584	2853
R_{int}	0.0529	0.0563	0.0329	0.0470	0.0234	0.0396
Parameters	199	275	217	380	199	217
Restraints	0	132	0	90	0	0
wR_2 (all data)	0.0517	0.0705	0.0453	0.0860	0.0368	0.0435
R_1	0.0218	0.0314	0.0185	0.0324	0.0210	0.0176
GooF	1.033	1.133	1.068	1.035	1.091	1.048

Table S2. Selected bond lengths (Å), angles, and dihedral angles (°) for **1**, **2**, **4**, and **5**·CH_2Cl_2. Atom labelling scheme as in Figures 1 and S4.

	**1**		**2**		**4**		**5**·CH_2Cl_2
Au1–N1	2.048(2)	Au1–N1	2.059(6)	Au1–N1	2.062(3)	Au1–N1	2.051(4)
Au1–N2	2.045(2)	Au1–N2	2.056(6)	Au1–N8	2.057(3)	Au1–N2	2.044(4)
Au1–Br1	2.3791(3)	Au1–Br1	2.3793(14)	Au1–Br1	2.3874(4)	Au1–Br1	2.3780(6)
Au1–Br2	2.3870(3)	Au1–Br2	2.3742(13)	Au1–Br2	2.3781(4)	Au1–Br2	2.3765(7)
N1–Au1–Br1	95.35(6)	N1–Au1–Br1	95.93(16)	N8–Au1–Br1	96.06(7)	N1–Au1–Br1	95.18(10)
Br1–Au1–Br2	88.597(10)	Br1–Au1–Br2	87.91(4)	Br1–Au1–Br2	88.405(14)	Br1–Au1–Br2	88.44(3)
N1–Au1–N2	80.95(9)	N1–Au1–N2	80.6(2)	N1–Au1–N8	81.21(11)	N1–Au1–N2	81.01(15)
N2–Au1–Br2	95.29(6)	N2–Au1–Br2	95.54(16)	N1–Au1–Br2	94.34(7)	N2–Au1–Br2	95.351(11)
C5–N1–Au1–Br1	171.5(2)	C6–N1–Au1–Br1	178.5(5)	C7–N8–Au1–Br1	175.7(2)	C1–N1–Au1–Br1	179.7(3)
C6–N2–Au1–Br2	176.7(2)	C7–N2–Au1–Br2	178.3(5)	C2–N1–Au1–Br2	172.7(2)	C9–N2–Au1–Br2	175.7(3)
N1–C5–C6–N2	3.9(4)	N1–C6–C7–N2	3.0(1)	N8–C7–C2–N1	1.8(4)	N1–C1–C9–N2	2.3(6)

Table S3. Selected bond lengths (Å), angles, and dihedral angles (°) for **6** and **9**. Atom labelling scheme as in Figure S5.

	**6**		**9**
Au1–N1	2.031(3)	Au1–N1	2.041(3)
Au1–N3	2.030(3)	Au1–N4	2.035(3)
Au1–Cl1	2.2645(9)	Au1–Cl1	2.2501(9)
Au1–Cl2	2.2656(10)	Au1–Cl2	2.2580(9)
N1–Au1–Cl2	95.22(9)	N1–Au1–Cl1	93.92(8)
Cl1–Au1–Cl2	88.69(4)	Cl1–Au1–Cl2	89.24(4)
N1–Au1–N3	80.94(11)	N1–Au1–N4	81.71(11)
N3–Au1–Cl1	95.61(8)	N4–Au1–Cl2	95.15(8)
C2–N1–Au1–Cl2	172.6(2)	C2–N1–Au1–Cl1	173.8(2)
C11–N3–Au1–Cl1	176.8(2)	C3–N4–Au1–Cl2	176.4(2)
N1–C2–C12–N3	0.6(4)	N1–C2–C3–N4	1.4(4)

Table S4. Optimized geometry calculated for the complex cation in compound **1** at DFT level (PBE0//def2-SVP/CRENBL) in the gas phase (total charge = +1, spin multiplicity = 1) in orthogonal Cartesian coordinate format (*Z* = atomic number).

**Atom number**	***Z***	**x**	**y**	**z**
1	79	0.000000	0.000000	0.885382
2	35	0.000000	1.657768	2.621788
3	35	0.000000	-1.657768	2.621788
4	7	0.000000	1.333792	-0.727895
5	7	0.000000	-1.333792	-0.727895
6	6	0.000000	0.736948	-1.943326
7	6	0.000000	1.513789	-3.099253
8	6	0.000000	2.902510	-2.992488
9	6	0.000000	3.488051	-1.731467
10	6	0.000000	2.664076	-0.611489
11	6	0.000000	-0.736948	-1.943326
12	6	0.000000	-1.513789	-3.099253
13	6	0.000000	-2.902510	-2.992488
14	6	0.000000	-3.488051	-1.731467
15	6	0.000000	-2.664076	-0.611489
16	1	0.000000	1.041741	-4.081451
17	1	0.000000	3.519903	-3.893572
18	1	0.000000	4.571398	-1.600959
19	1	0.000000	3.059548	0.408621
20	1	0.000000	-1.041741	-4.081451
21	1	0.000000	-3.519903	-3.893572
22	1	0.000000	-4.571398	-1.600959
23	1	0.000000	-3.059548	0.408621

Table S5. Optimized geometry calculated for the complex cation in compound **1** at DFT level (PBE0//def2-SVP/CRENBL) in water (SCRF IEF-PCM; total charge = +1, spin multiplicity = 1) in orthogonal Cartesian coordinate format (Z = atomic number).

Atom number	Z	x	y	z
1	79	0.000000	0.000000	0.863762
2	35	0.000000	1.669323	2.615712
3	35	0.000000	-1.669323	2.615712
4	7	0.000000	1.329981	-0.726773
5	7	0.000000	-1.329981	-0.726773
6	6	0.000000	0.734440	-1.943437
7	6	0.000000	1.510912	-3.098097
8	6	0.000000	2.898418	-2.989848
9	6	0.000000	3.482784	-1.728757
10	6	0.000000	2.659997	-0.609479
11	6	0.000000	-0.734440	-1.943437
12	6	0.000000	-1.510912	-3.098097
13	6	0.000000	-2.898418	-2.989848
14	6	0.000000	-3.482784	-1.728757
15	6	0.000000	-2.659997	-0.609479
16	1	0.000000	1.038313	-4.079273
17	1	0.000000	3.515147	-3.890312
18	1	0.000000	4.564994	-1.596125
19	1	0.000000	3.061936	0.405708
20	1	0.000000	-1.038313	-4.079273
21	1	0.000000	-3.515147	-3.890312
22	1	0.000000	-4.564994	-1.596125
23	1	0.000000	-3.061936	0.405708

Table S6. Optimized geometry calculated for the complex cation in compound **2** at DFT level (PBE0//def2-SVP/CRENBL) in the gas phase (total charge = +1, spin multiplicity = 1) in orthogonal Cartesian coordinate format (*Z* = atomic number).

Atom number	*Z*	x	y	z
1	6	0.000000	2.660388	0.067356
2	7	0.000000	1.329792	-0.038882
3	6	0.000000	0.737626	-1.258207
4	6	0.000000	1.514037	-2.409185
5	6	0.000000	2.915078	-2.331815
6	6	0.000000	3.479126	-1.053272
7	79	0.000000	0.000000	1.569287
8	35	0.000000	-1.662219	3.303264
9	6	0.000000	-0.737626	-1.258207
10	7	0.000000	-1.329792	-0.038882
11	6	0.000000	-2.660388	0.067356
12	6	0.000000	-3.479126	-1.053272
13	6	0.000000	-2.915078	-2.331815
14	6	0.000000	-1.514037	-2.409185
15	6	0.000000	-3.750580	-3.568699
16	6	0.000000	3.750580	-3.568699
17	35	0.000000	1.662219	3.303264
18	1	0.000000	1.032633	-3.387815
19	1	0.000000	4.562034	-0.915839
20	1	0.000000	3.063478	1.084377
21	1	0.000000	-1.032633	-3.387815
22	1	0.000000	-4.562034	-0.915839
23	1	0.000000	-3.063478	1.084377
24	1	0.885651	3.529339	-4.185108
25	1	-0.885651	3.529339	-4.185108
26	1	0.000000	4.823042	-3.336927
27	1	0.885651	-3.529339	-4.185108
28	1	-0.885651	-3.529339	-4.185108
29	1	0.000000	-4.823042	-3.336927

Table S7. Optimized geometry calculated for the complex cation in compound **2** at DFT level (PBE0//def2-SVP/CRENBL) in water (SCRF IEF-PCM; total charge = +1, spin multiplicity = 1) in orthogonal Cartesian coordinate format (*Z* = atomic number).

Atom number	*Z*	x	y	z
1	6	0.000000	2.656654	0.068130
2	7	0.000000	1.326126	-0.039805
3	6	0.000000	0.735171	-1.259894
4	6	0.000000	1.510815	-2.410154
5	6	0.000000	2.910145	-2.329578
6	6	0.000000	3.474630	-1.051617
7	79	0.000000	0.000000	1.547248
8	35	0.000000	-1.672365	3.297487
9	6	0.000000	-0.735171	-1.259894
10	7	0.000000	-1.326126	-0.039805
11	6	0.000000	-2.656654	0.068130
12	6	0.000000	-3.474630	-1.051617
13	6	0.000000	-2.910145	-2.329578
14	6	0.000000	-1.510815	-2.410154
15	6	0.000000	-3.745076	-3.565896
16	6	0.000000	3.745076	-3.565896
17	35	0.000000	1.672365	3.297487
18	1	0.000000	1.029219	-3.387874
19	1	0.000000	4.556614	-0.912821
20	1	0.000000	3.066693	1.079815
21	1	0.000000	-1.029219	-3.387874
22	1	0.000000	-4.556614	-0.912821
23	1	0.000000	-3.066693	1.079815
24	1	0.884362	3.516647	-4.180247
25	1	-0.884362	3.516647	-4.180247
26	1	0.000000	4.816655	-3.332142
27	1	0.884362	-3.516647	-4.180247
28	1	-0.884362	-3.516647	-4.180247
29	1	0.000000	-4.816655	-3.332142

Table S8. Optimized geometry calculated for the complex cation in compound **3** at DFT level (PBE0//def2-SVP/CRENBL) in the gas phase (total charge = +1, spin multiplicity = 1) in orthogonal Cartesian coordinate format (*Z* = atomic number).

Atom number	*Z*	x	y	z
1	79	0.000000	0.000000	-2.072483
2	35	1.664292	0.000000	-3.805791
3	35	-1.664292	0.000000	-3.805791
4	7	1.328319	0.000000	-0.466384
5	7	-1.328319	0.000000	-0.466384
6	6	0.737493	0.000000	0.753542
7	6	1.514726	0.000000	1.902835
8	6	2.917906	0.000000	1.836538
9	6	3.476403	0.000000	0.554536
10	6	2.658252	0.000000	-0.567400
11	6	-0.737493	0.000000	0.753542
12	6	-1.514726	0.000000	1.902835
13	6	-2.917906	0.000000	1.836538
14	6	-3.476403	0.000000	0.554536
15	6	-2.658252	0.000000	-0.567400
16	1	1.021291	0.000000	2.874114
17	1	4.555134	0.000000	0.402790
18	1	3.064987	0.000000	-1.582802
19	1	-1.021291	0.000000	2.874114
20	1	-4.555134	0.000000	0.402790
21	1	-3.064987	0.000000	-1.582802
22	6	-3.749512	0.000000	3.113306
23	6	3.749512	0.000000	3.113306
24	6	5.249943	0.000000	2.814672
25	1	5.561390	0.894347	2.253473
26	1	5.809942	0.000000	3.760737
27	1	5.561390	-0.894347	2.253473
28	6	-5.249943	0.000000	2.814672
29	1	-5.561390	-0.894347	2.253473
30	1	-5.809942	0.000000	3.760737
31	1	-5.561390	0.894347	2.253473
32	6	-3.402706	1.259803	3.925838
33	1	-4.001064	1.278462	4.848944
34	1	-2.343193	1.290136	4.222305
35	1	-3.626367	2.177079	3.360394
36	6	-3.402706	-1.259803	3.925838
37	1	-3.626367	-2.177079	3.360394
38	1	-2.343193	-1.290136	4.222305
39	1	-4.001064	-1.278462	4.848944
40	6	3.402706	-1.259803	3.925838
41	1	2.343193	-1.290136	4.222305
42	1	3.626367	-2.177079	3.360394
43	1	4.001064	-1.278462	4.848944
44	6	3.402706	1.259803	3.925838
45	1	3.626367	2.177079	3.360394
46	1	2.343193	1.290136	4.222305
47	1	4.001064	1.278462	4.848944

Table S9. Optimized geometry calculated for the complex cation in compound **3** at DFT level (PBE0//def2-SVP/CRENBL) in water (SCRF IEF-PCM; total charge = +1, spin multiplicity = 1) in orthogonal Cartesian coordinate format (*Z* = atomic number).

Atom number	*Z*	x	y	z
1	6	0.000000	2.655143	0.081983
2	7	0.000000	1.325701	-0.020331
3	6	0.000000	0.735213	-1.240579
4	6	0.000000	1.511446	-2.389378
5	6	0.000000	2.912956	-2.319852
6	6	0.000000	3.472803	-1.039528
7	79	0.000000	0.000000	1.566779
8	35	0.000000	-1.674566	3.315902
9	6	0.000000	-0.735213	-1.240579
10	7	0.000000	-1.325701	-0.020331
11	6	0.000000	-2.655143	0.081983
12	6	0.000000	-3.472803	-1.039528
13	6	0.000000	-2.912956	-2.319852
14	6	0.000000	-1.511446	-2.389378
15	6	0.000000	-3.742877	-3.597235
16	6	0.000000	3.742877	-3.597235
17	35	0.000000	1.674566	3.315902
18	1	0.000000	1.015017	-3.358724
19	1	0.000000	4.550393	-0.883526
20	1	0.000000	3.068728	1.092466
21	1	0.000000	-1.015017	-3.358724
22	1	0.000000	-4.550393	-0.883526
23	1	0.000000	-3.068728	1.092466
24	6	0.000000	-5.243425	-3.300654
25	6	-1.259109	-3.395025	-4.409573
26	6	1.259109	-3.395025	-4.409573
27	6	0.000000	5.243425	-3.300654
28	6	1.259109	3.395025	-4.409573
29	6	-1.259109	3.395025	-4.409573
30	1	-1.272988	-3.989458	-5.335649
31	1	-2.173658	-3.624696	-3.842077
32	1	-1.290396	-2.333238	-4.695757
33	1	0.000000	-5.799656	-4.249319
34	1	0.893553	-5.551938	-2.736855
35	1	-0.893553	-5.551938	-2.736855
36	1	1.272988	-3.989458	-5.335649
37	1	1.290396	-2.333238	-4.695757
38	1	2.173658	-3.624696	-3.842077
39	1	-1.272988	3.989458	-5.335649
40	1	-1.290396	2.333238	-4.695757
41	1	-2.173658	3.624696	-3.842077
42	1	1.272988	3.989458	-5.335649
43	1	2.173658	3.624696	-3.842077
44	1	1.290396	2.333238	-4.695757
45	1	0.000000	5.799656	-4.249319
46	1	-0.893553	5.551938	-2.736855
47	1	0.893553	5.551938	-2.736855

Table S10. Optimized geometry calculated for the complex cation in compound **4** at DFT level (PBE0//def2-SVP/CRENBL) in the gas phase (total charge = +1, spin multiplicity = 1) in orthogonal Cartesian coordinate format (Z = atomic number).

Atom number	Z	x	y	z
1	6	0.000000	2.671944	0.066189
2	7	0.000000	1.348256	0.006497
3	6	0.000000	0.713708	-1.194611
4	6	0.000000	1.421449	-2.413383
5	6	0.000000	2.832707	-2.335486
6	6	0.000000	3.451116	-1.101732
7	79	0.000000	0.000000	1.612870
8	35	0.000002	-1.673128	3.331965
9	6	0.000000	-0.713708	-1.194611
10	7	0.000000	-1.348256	0.006497
11	6	0.000000	-2.671944	0.066189
12	6	0.000000	-3.451116	-1.101732
13	6	0.000000	-2.832707	-2.335486
14	6	0.000000	-1.421449	-2.413383
15	35	-0.000002	1.673128	3.331965
16	1	0.000000	4.538553	-1.011369
17	1	0.000000	3.115151	1.066993
18	1	0.000000	-4.538553	-1.011369
19	1	0.000000	-3.115151	1.066993
20	1	0.000000	3.426045	-3.253760
21	1	0.000000	-3.426045	-3.253760
22	6	0.000000	0.681937	-3.640957
23	1	0.000000	1.234500	-4.583330
24	6	0.000000	-0.681937	-3.640957
25	1	0.000000	-1.234500	-4.583330

Table S11. Optimized geometry calculated for the complex cation in compound **4** at DFT level (PBE0//def2-SVP/CRENBL) in water (SCRF IEF-PCM; total charge = +1, spin multiplicity = 1) in orthogonal Cartesian coordinate format (*Z* = atomic number).

Atom number	*Z*	x	y	z
1	6	0.000009	2.667660	0.067616
2	7	0.000006	1.344680	0.006581
3	6	0.000003	0.711474	-1.195786
4	6	0.000005	1.421313	-2.411868
5	6	0.000008	2.831620	-2.333764
6	6	0.000010	3.447006	-1.099081
7	79	0.000000	0.000000	1.591921
8	35	0.000135	-1.685007	3.325702
9	6	-0.000003	-0.711474	-1.195786
10	7	-0.000006	-1.344680	0.006581
11	6	-0.000009	-2.667660	0.067616
12	6	-0.000010	-3.447006	-1.099081
13	6	-0.000008	-2.831620	-2.333764
14	6	-0.000005	-1.421313	-2.411868
15	35	-0.000135	1.685007	3.325702
16	1	0.000013	4.533272	-1.005072
17	1	0.000011	3.117468	1.063229
18	1	-0.000013	-4.533272	-1.005072
19	1	-0.000011	-3.117468	1.063229
20	1	0.000010	3.424046	-3.251133
21	1	-0.000010	-3.424046	-3.251133
22	6	0.000002	0.681823	-3.639192
23	1	0.000004	1.235948	-4.579739
24	6	-0.000002	-0.681823	-3.639192
25	1	-0.000004	-1.235948	-4.579739

Table S12. Optimized geometry calculated for the complex cation in compound **5** at DFT level (PBE0//def2-SVP/CRENBL) in the gas phase (total charge = +1, spin multiplicity = 1) in orthogonal Cartesian coordinate format (*Z* = atomic number).

Atom number	*Z*	x	y	z
1	79	-0.044868	2.619775	0.000000
2	7	0.010719	1.019834	1.335864
3	7	0.010719	1.019834	-1.335864
4	6	0.037887	1.085251	2.659839
5	1	0.018769	2.087110	3.099339
6	6	0.064888	-0.069460	3.444156
7	1	0.049527	0.037231	4.529924
8	6	0.058906	-1.335020	2.863877
9	6	0.072095	-1.402457	1.433999
10	6	0.184638	-2.616105	0.682398
11	1	0.302150	-3.554900	1.224600
12	6	0.184638	-2.616105	-0.682398
13	1	0.302150	-3.554900	-1.224600
14	6	0.072095	-1.402457	-1.433999
15	6	0.058906	-1.335020	-2.863877
16	6	0.064888	-0.069460	-3.444156
17	1	0.049527	0.037231	-4.529924
18	6	0.037887	1.085251	-2.659839
19	1	0.018769	2.087110	-3.099339
20	6	0.033136	-0.190073	-0.715031
21	6	0.033136	-0.190073	0.715031
22	6	0.041580	-2.535808	3.718944
23	6	0.944661	-2.641727	4.788946
24	1	1.691288	-1.859983	4.950132
25	6	0.917530	-3.754176	5.624122
26	1	1.636205	-3.834504	6.442570
27	6	-0.024350	-4.761712	5.417190
28	1	-0.050053	-5.630142	6.079287
29	6	-0.937807	-4.656970	4.368269
30	1	-1.689067	-5.435102	4.216274
31	6	-0.902829	-3.556325	3.517402
32	1	-1.641793	-3.468521	2.717057
33	6	0.041580	-2.535808	-3.718944
34	6	0.944661	-2.641727	-4.788946
35	1	1.691288	-1.859983	-4.950132
36	6	0.917530	-3.754176	-5.624122
37	1	1.636205	-3.834504	-6.442570
38	6	-0.024350	-4.761712	-5.417190
39	1	-0.050053	-5.630142	-6.079287
40	6	-0.937807	-4.656970	-4.368269
41	1	-1.689067	-5.435102	-4.216274
42	6	-0.902829	-3.556325	-3.517402
43	1	-1.641793	-3.468521	-2.717057
44	35	-0.101361	4.340462	-1.675880
45	35	-0.101361	4.340462	1.675880

Table S13. Optimized geometry calculated for the complex cation in compound **5** at DFT level (PBE0//def2-SVP/CRENBL) in water (SCRF IEF-PCM; total charge = +1, spin multiplicity = 1) in orthogonal Cartesian coordinate format (Z = atomic number).

Atom number	Z	x	y	z
1	79	-0.021480	2.602697	0.000000
2	7	0.004928	1.017651	1.333576
3	7	0.004928	1.017651	-1.333576
4	6	0.025747	1.085492	2.656340
5	1	0.010780	2.082484	3.102534
6	6	0.047352	-0.069070	3.441546
7	1	0.032706	0.043094	4.526149
8	6	0.045785	-1.332163	2.860985
9	6	0.060887	-1.403408	1.433384
10	6	0.167209	-2.617174	0.682031
11	1	0.276115	-3.559134	1.219439
12	6	0.167209	-2.617174	-0.682031
13	1	0.276115	-3.559134	-1.219439
14	6	0.060887	-1.403408	-1.433384
15	6	0.045785	-1.332163	-2.860985
16	6	0.047352	-0.069070	-3.441546
17	1	0.032706	0.043094	-4.526149
18	6	0.025747	1.085492	-2.656340
19	1	0.010780	2.082484	-3.102534
20	6	0.025287	-0.192342	-0.713504
21	6	0.025287	-0.192342	0.713504
22	6	0.034142	-2.533417	3.719678
23	6	0.942896	-2.634400	4.784877
24	1	1.682517	-1.845914	4.943431
25	6	0.925229	-3.747092	5.621458
26	1	1.647663	-3.821301	6.437396
27	6	-0.010509	-4.761749	5.418023
28	1	-0.026613	-5.631839	6.078530
29	6	-0.928450	-4.661490	4.372180
30	1	-1.672508	-5.446084	4.217538
31	6	-0.905252	-3.558365	3.522578
32	1	-1.643568	-3.477992	2.721394
33	6	0.034142	-2.533417	-3.719678
34	6	0.942896	-2.634400	-4.784877
35	1	1.682517	-1.845914	-4.943431
36	6	0.925229	-3.747092	-5.621458
37	1	1.647663	-3.821301	-6.437396
38	6	-0.010509	-4.761749	-5.418023
39	1	-0.026613	-5.631839	-6.078530
40	6	-0.928450	-4.661490	-4.372180
41	1	-1.672508	-5.446084	-4.217538
42	6	-0.905252	-3.558365	-3.522578
43	1	-1.643568	-3.477992	-2.721394
44	35	-0.037321	4.339655	-1.684045
45	35	-0.037321	4.339655	1.684045

Table S14. Optimized geometry calculated for the complex cation in compound **6** at DFT level (PBE0//def2-SVP/CRENBL) in the gas phase (total charge = +1, spin multiplicity = 1) in orthogonal Cartesian coordinate format (Z = atomic number).

Atom number	*Z*	x	y	z
1	79	0.000000	0.000000	0.858044
2	17	0.000000	1.594416	2.477569
3	17	0.000000	-1.594416	2.477569
4	7	0.000000	1.327160	-0.724028
5	7	0.000000	-1.327160	-0.724028
6	6	0.000000	0.736670	-1.944212
7	6	0.000000	1.522246	-3.092992
8	6	0.000000	2.910564	-2.973005
9	6	0.000000	3.487016	-1.707464
10	6	0.000000	2.656438	-0.592262
11	6	0.000000	-0.736670	-1.944212
12	6	0.000000	-1.522246	-3.092992
13	6	0.000000	-2.910564	-2.973005
14	6	0.000000	-3.487016	-1.707464
15	6	0.000000	-2.656438	-0.592262
16	1	0.000000	1.057579	-4.078966
17	1	0.000000	3.535663	-3.868806
18	1	0.000000	4.569513	-1.569610
19	1	0.000000	3.036327	0.433759
20	1	0.000000	-1.057579	-4.078966
21	1	0.000000	-3.535663	-3.868806
22	1	0.000000	-4.569513	-1.569610
23	1	0.000000	-3.036327	0.433759

Table S15. Optimized geometry calculated for the complex cation in compound **6** at DFT level (PBE0//def2-SVP/CRENBL) in water (SCRF IEF-PCM; total charge = +1, spin multiplicity = 1) in orthogonal Cartesian coordinate format (*Z* = atomic number).

Atom number	*Z*	x	y	z
1	79	0.000000	0.000000	0.846792
2	17	0.000000	1.601698	2.486983
3	17	0.000000	-1.601698	2.486983
4	7	0.000000	1.322736	-0.717788
5	7	0.000000	-1.322736	-0.717788
6	6	0.000000	0.733867	-1.938860
7	6	0.000000	1.519102	-3.086430
8	6	0.000000	2.906111	-2.964888
9	6	0.000000	3.481276	-1.699303
10	6	0.000000	2.651540	-0.585144
11	6	0.000000	-0.733867	-1.938860
12	6	0.000000	-1.519102	-3.086430
13	6	0.000000	-2.906111	-2.964888
14	6	0.000000	-3.481276	-1.699303
15	6	0.000000	-2.651540	-0.585144
16	1	0.000000	1.054025	-4.071415
17	1	0.000000	3.530596	-3.859992
18	1	0.000000	4.562574	-1.559361
19	1	0.000000	3.037721	0.436049
20	1	0.000000	-1.054025	-4.071415
21	1	0.000000	-3.530596	-3.859992
22	1	0.000000	-4.562574	-1.559361
23	1	0.000000	-3.037721	0.436049

Table S16. Optimized geometry calculated for the complex cation in compound **7** at DFT level (PBE0//def2-SVP/CRENBL) in the gas phase (total charge = +1, spin multiplicity = 1) in orthogonal Cartesian coordinate format (*Z* = atomic number).

Atom number	*Z*	x	y	z
1	79	0.000000	0.000000	1.193790
2	17	0.000000	1.598315	2.812059
3	17	0.000000	-1.598315	2.812059
4	7	0.000000	1.323145	-0.385481
5	7	0.000000	-1.323145	-0.385481
6	6	0.000000	0.737135	-1.608792
7	6	0.000000	1.521813	-2.753179
8	6	0.000000	2.922775	-2.663142
9	6	0.000000	3.477952	-1.380062
10	6	0.000000	2.652952	-0.264097
11	6	0.000000	-0.737135	-1.608792
12	6	0.000000	-1.521813	-2.753179
13	6	0.000000	-2.922775	-2.663142
14	6	0.000000	-3.477952	-1.380062
15	6	0.000000	-2.652952	-0.264097
16	1	0.000000	1.047244	-3.735369
17	1	0.000000	4.560001	-1.235687
18	1	0.000000	3.040827	0.758779
19	1	0.000000	-1.047244	-3.735369
20	1	0.000000	-4.560001	-1.235687
21	1	0.000000	-3.040827	0.758779
22	6	0.000000	-3.768416	-3.893102
23	1	-0.885518	-3.551770	-4.510985
24	1	0.885518	-3.551770	-4.510985
25	1	0.000000	-4.838815	-3.652840
26	6	0.000000	3.768416	-3.893102
27	1	0.885518	3.551770	-4.510985
28	1	-0.885518	3.551770	-4.510985
29	1	0.000000	4.838815	-3.652840

Table S17. Optimized geometry calculated for the complex cation in compound **7** at DFT level (PBE0//def2-SVP/CRENBL) in water (SCRF IEF-PCM; total charge = +1, spin multiplicity = 1) in orthogonal Cartesian coordinate format (*Z* = atomic number).

Atom number	*Z*	x	y	z
1	79	0.000000	0.000000	-1.194760
2	17	-1.604579	0.000000	-2.834728
3	17	1.604579	0.000000	-2.834728
4	7	-1.318967	0.000000	0.366605
5	7	1.318967	0.000000	0.366605
6	6	-0.734662	0.000000	1.590995
7	6	-1.518987	0.000000	2.734277
8	6	-2.918238	0.000000	2.640463
9	6	-3.473274	0.000000	1.357879
10	6	-2.648332	0.000000	0.243396
11	6	0.734662	0.000000	1.590995
12	6	1.518987	0.000000	2.734277
13	6	2.918238	0.000000	2.640463
14	6	3.473274	0.000000	1.357879
15	6	2.648332	0.000000	0.243396
16	1	-1.044835	0.000000	3.715832
17	1	-4.554273	0.000000	1.211594
18	1	-3.042568	0.000000	-0.774498
19	1	1.044835	0.000000	3.715832
20	1	4.554273	0.000000	1.211594
21	1	3.042568	0.000000	-0.774498
22	6	3.763516	0.000000	3.869482
23	1	3.539980	0.884372	4.485663
24	1	3.539980	-0.884372	4.485663
25	1	4.833097	0.000000	3.626946
26	6	-3.763516	0.000000	3.869482
27	1	-3.539980	-0.884372	4.485663
28	1	-3.539980	0.884372	4.485663
29	1	-4.833097	0.000000	3.626946

Table S18. Optimized geometry calculated for the complex cation in compound **8** at DFT level (PBE0//def2-SVP/CRENBL) in the gas phase (total charge = +1, spin multiplicity = 1) in orthogonal Cartesian coordinate format (*Z* = atomic number).

Atom number	*Z*	x	y	z
1	79	0.000000	0.000000	-2.033012
2	17	1.599979	0.000000	-3.651294
3	17	-1.599979	0.000000	-3.651294
4	7	1.321848	0.000000	-0.455769
5	7	-1.321848	0.000000	-0.455769
6	6	0.737030	0.000000	0.768375
7	6	1.522448	0.000000	1.910889
8	6	2.925784	0.000000	1.832036
9	6	3.475387	0.000000	0.545736
10	6	2.650992	0.000000	-0.571615
11	6	-0.737030	0.000000	0.768375
12	6	-1.522448	0.000000	1.910889
13	6	-2.925784	0.000000	1.832036
14	6	-3.475387	0.000000	0.545736
15	6	-2.650992	0.000000	-0.571615
16	1	1.035876	0.000000	2.885826
17	1	4.553175	0.000000	0.387314
18	1	3.042632	0.000000	-1.592898
19	1	-1.035876	0.000000	2.885826
20	1	-4.553175	0.000000	0.387314
21	1	-3.042632	0.000000	-1.592898
22	6	-3.767106	0.000000	3.101996
23	6	3.767106	0.000000	3.101996
24	6	5.265309	0.000000	2.792129
25	1	5.572731	0.894410	2.228843
26	1	5.832078	0.000000	3.734086
27	1	5.572731	-0.894410	2.228843
28	6	-5.265309	0.000000	2.792129
29	1	-5.572731	-0.894410	2.228843
30	1	-5.832078	0.000000	3.734086
31	1	-5.572731	0.894410	2.228843
32	6	-3.425973	1.259966	3.916999
33	1	-4.031831	1.278815	4.835136
34	1	-2.368955	1.289741	4.222266
35	1	-3.644875	2.177258	3.349784
36	6	-3.425973	-1.259966	3.916999
37	1	-3.644875	-2.177258	3.349784
38	1	-2.368955	-1.289741	4.222266
39	1	-4.031831	-1.278815	4.835136
40	6	3.425973	-1.259966	3.916999
41	1	2.368955	-1.289741	4.222266
42	1	3.644875	-2.177258	3.349784
43	1	4.031831	-1.278815	4.835136
44	6	3.425973	1.259966	3.916999
45	1	3.644875	2.177258	3.349784
46	1	2.368955	1.289741	4.222266
47	1	4.031831	1.278815	4.835136

Table S19. Optimized geometry calculated for the complex cation in compound **8** at DFT level (PBE0//def2-SVP/CRENBL) in water (SCRF IEF-PCM; total charge = +1, spin multiplicity = 1) in orthogonal Cartesian coordinate format (*Z* = atomic number).

Atom number	*Z*	x	y	z
1	79	0.000000	0.000000	-2.014702
2	17	1.606007	0.000000	-3.653609
3	17	-1.606007	0.000000	-3.653609
4	7	1.318543	0.000000	-0.453138
5	7	-1.318543	0.000000	-0.453138
6	6	0.734620	0.000000	0.771133
7	6	1.519171	0.000000	1.913194
8	6	2.920570	0.000000	1.830770
9	6	3.471351	0.000000	0.546054
10	6	2.646985	0.000000	-0.570447
11	6	-0.734620	0.000000	0.771133
12	6	-1.519171	0.000000	1.913194
13	6	-2.920570	0.000000	1.830770
14	6	-3.471351	0.000000	0.546054
15	6	-2.646985	0.000000	-0.570447
16	1	1.029717	0.000000	2.886230
17	1	4.547865	0.000000	0.383091
18	1	3.045528	0.000000	-1.586756
19	1	-1.029717	0.000000	2.886230
20	1	-4.547865	0.000000	0.383091
21	1	-3.045528	0.000000	-1.586756
22	6	-3.760265	0.000000	3.101663
23	6	3.760265	0.000000	3.101663
24	6	5.258743	0.000000	2.794495
25	1	5.563475	0.893611	2.228747
26	1	5.821415	0.000000	3.739340
27	1	5.563475	-0.893611	2.228747
28	6	-5.258743	0.000000	2.794495
29	1	-5.563475	-0.893611	2.228747
30	1	-5.821415	0.000000	3.739340
31	1	-5.563475	0.893611	2.228747
32	6	-3.417972	1.259215	3.916377
33	1	-4.019460	1.273168	4.837857
34	1	-2.358431	1.290265	4.210741
35	1	-3.643103	2.173790	3.347125
36	6	-3.417972	-1.259215	3.916377
37	1	-3.643103	-2.173790	3.347125
38	1	-2.358431	-1.290265	4.210741
39	1	-4.019460	-1.273168	4.837857
40	6	3.417972	-1.259215	3.916377
41	1	2.358431	-1.290265	4.210741
42	1	3.643103	-2.173790	3.347125
43	1	4.019460	-1.273168	4.837857
44	6	3.417972	1.259215	3.916377
45	1	3.643103	2.173790	3.347125
46	1	2.358431	1.290265	4.210741
47	1	4.019460	1.273168	4.837857

Table S20. Optimized geometry calculated for the complex cation in compound **9** at DFT level (PBE0//def2-SVP/CRENBL) in the gas phase (total charge = +1, spin multiplicity = 1) in orthogonal Cartesian coordinate format (*Z* = atomic number).

Atom number	*Z*	x	y	z
1	79	0.000000	0.000000	1.150930
2	17	-1.609458	0.000000	2.754324
3	17	1.609458	0.000000	2.754324
4	7	-1.341890	0.000000	-0.426016
5	7	1.341890	0.000000	-0.426016
6	6	-2.665155	0.000000	-0.355282
7	1	-3.097460	0.000000	0.650110
8	6	-3.447761	0.000000	-1.521571
9	1	-4.534814	0.000000	-1.426568
10	6	-2.835442	0.000000	-2.759138
11	6	-1.424165	0.000000	-2.845269
12	6	-0.682304	0.000000	-4.072412
13	1	-1.233336	0.000000	-5.015381
14	6	0.682304	0.000000	-4.072412
15	1	1.233336	0.000000	-5.015381
16	6	1.424165	0.000000	-2.845269
17	6	2.835442	0.000000	-2.759138
18	6	3.447761	0.000000	-1.521571
19	1	4.534814	0.000000	-1.426568
20	6	2.665155	0.000000	-0.355282
21	1	3.097460	0.000000	0.650110
22	6	0.712440	0.000000	-1.630713
23	6	-0.712440	0.000000	-1.630713
24	1	3.433598	0.000000	-3.674023
25	1	-3.433598	0.000000	-3.674023

Table S21. Optimized geometry calculated for the complex cation in compound **9** at DFT level (PBE0//def2-SVP/CRENBL) in water (SCRF IEF-PCM; total charge = +1, spin multiplicity = 1) in orthogonal Cartesian coordinate format (*Z* = atomic number).

Atom number	*Z*	x	y	z
1	79	0.000000	0.000000	1.132925
2	17	-1.616190	0.000000	2.756292
3	17	1.616190	0.000000	2.756292
4	7	-1.338156	0.000000	-0.426643
5	7	1.338156	0.000000	-0.426643
6	6	-2.660864	0.000000	-0.354769
7	1	-3.099852	0.000000	0.645323
8	6	-3.443091	0.000000	-1.519833
9	1	-4.529024	0.000000	-1.422183
10	6	-2.833323	0.000000	-2.758201
11	6	-1.423118	0.000000	-2.844650
12	6	-0.682228	0.000000	-4.071732
13	1	-1.235398	0.000000	-5.012812
14	6	0.682228	0.000000	-4.071732
15	1	1.235398	0.000000	-5.012812
16	6	1.423118	0.000000	-2.844650
17	6	2.833323	0.000000	-2.758201
18	6	3.443091	0.000000	-1.519833
19	1	4.529024	0.000000	-1.422183
20	6	2.660864	0.000000	-0.354769
21	1	3.099852	0.000000	0.645323
22	6	0.710338	0.000000	-1.631840
23	6	-0.710338	0.000000	-1.631840
24	1	3.431374	0.000000	-3.671890
25	1	-3.431374	0.000000	-3.671890

Table S22. Optimized geometry calculated for the complex cation in compound **10** at DFT level (PBE0//def2-SVP/CRENBL) in the gas phase (total charge = +1, spin multiplicity = 1) in orthogonal Cartesian coordinate format (*Z* = atomic number).

Atom number	*Z*	x	y	z
1	79	-0.130721	2.611696	0.000000
2	17	-0.219909	4.215682	1.611397
3	17	-0.219909	4.215682	-1.611397
4	7	-0.040703	1.041351	1.330244
5	7	-0.040703	1.041351	-1.330244
6	6	-0.013750	1.117654	2.654221
7	1	-0.053349	2.123473	3.082797
8	6	0.037518	-0.034700	3.440848
9	1	0.019864	0.075479	4.526241
10	6	0.056801	-1.303746	2.866009
11	6	0.071361	-1.378589	1.435796
12	6	0.209868	-2.589224	0.682806
13	1	0.347987	-3.525758	1.224116
14	6	0.209868	-2.589224	-0.682806
15	1	0.347987	-3.525758	-1.224116
16	6	0.071361	-1.378589	-1.435796
17	6	0.056801	-1.303746	-2.866009
18	6	0.037518	-0.034700	-3.440848
19	1	0.019864	0.075479	-4.526241
20	6	-0.013750	1.117654	-2.654221
21	1	-0.053349	2.123473	-3.082797
22	6	0.006729	-0.170731	-0.714041
23	6	0.006729	-0.170731	0.714041
24	6	0.062665	-2.500230	3.726033
25	6	0.958634	-2.578985	4.804609
26	1	1.684331	-1.778446	4.969061
27	6	0.952124	-3.688509	5.643880
28	1	1.665391	-3.747654	6.468812
29	6	0.037597	-4.720206	5.432871
30	1	0.027851	-5.586305	6.098436
31	6	-0.869082	-4.642742	4.375674
32	1	-1.599482	-5.439905	4.220804
33	6	-0.854058	-3.545196	3.520424
34	1	-1.588828	-3.478836	2.714190
35	6	0.062665	-2.500230	-3.726033
36	6	0.958634	-2.578985	-4.804609
37	1	1.684331	-1.778446	-4.969061
38	6	0.952124	-3.688509	-5.643880
39	1	1.665391	-3.747654	-6.468812
40	6	0.037597	-4.720206	-5.432871
41	1	0.027851	-5.586305	-6.098436
42	6	-0.869082	-4.642742	-4.375674
43	1	-1.599482	-5.439905	-4.220804
44	6	-0.854058	-3.545196	-3.520424
45	1	-1.588828	-3.478836	-2.714190

Table S23. Optimized geometry calculated for the complex cation in compound **10** at DFT level (PBE0//def2-SVP/CRENBL) in water (SCRF IEF-PCM; total charge = +1, spin multiplicity = 1) in orthogonal Cartesian coordinate format (*Z* = atomic number).

Atom number	*Z*	x	y	z
1	79	-0.019607	2.566287	0.000000
2	17	-0.039161	4.193033	1.615603
3	17	-0.039161	4.193033	-1.615603
4	7	0.008610	1.006805	1.327179
5	7	0.008610	1.006805	-1.327179
6	6	0.031008	1.085178	2.649659
7	1	0.017066	2.086804	3.084754
8	6	0.052202	-0.067558	3.437654
9	1	0.038096	0.048634	4.521820
10	6	0.048719	-1.334258	2.862812
11	6	0.062882	-1.413576	1.435040
12	6	0.167992	-2.627181	0.682416
13	1	0.276484	-3.569691	1.218949
14	6	0.167992	-2.627181	-0.682416
15	1	0.276484	-3.569691	-1.218949
16	6	0.062882	-1.413576	-1.435040
17	6	0.048719	-1.334258	-2.862812
18	6	0.052202	-0.067558	-3.437654
19	1	0.038096	0.048634	-4.521820
20	6	0.031008	1.085178	-2.649659
21	1	0.017066	2.086804	-3.084754
22	6	0.027709	-0.206100	-0.712308
23	6	0.027709	-0.206100	0.712308
24	6	0.034681	-2.531069	3.726745
25	6	0.936707	-2.625383	4.798411
26	1	1.673502	-1.834640	4.958656
27	6	0.916555	-3.735040	5.638817
28	1	1.633912	-3.804447	6.459617
29	6	-0.015183	-4.752962	5.433006
30	1	-0.033216	-5.620615	6.096660
31	6	-0.926633	-4.659200	4.380902
32	1	-1.667639	-5.446309	4.224539
33	6	-0.900738	-3.559357	3.527246
34	1	-1.634502	-3.483809	2.721473
35	6	0.034681	-2.531069	-3.726745
36	6	0.936707	-2.625383	-4.798411
37	1	1.673502	-1.834640	-4.958656
38	6	0.916555	-3.735040	-5.638817
39	1	1.633912	-3.804447	-6.459617
40	6	-0.015183	-4.752962	-5.433006
41	1	-0.033216	-5.620615	-6.096660
42	6	-0.926633	-4.659200	-4.380902
43	1	-1.667639	-5.446309	-4.224539
44	6	-0.900738	-3.559357	-3.527246
45	1	-1.634502	-3.483809	-2.721473

Table S24. Selected Optimized bond lengths (Å) and angles (°) calculated at DFT level (PBE0//def2-SVP/CRENBL) in the gas phase and in water solution (SCRF IEF-PCM; values in parentheses) for the complex cations in compounds **1**–**10**; numbering scheme as in Figure S9; X = Br (**1**–**5**), Cl (**6**–**10**).

	**1**	**2**	**3**	**4**	**5**	**6**	**7**	**8**	**9**	**10**
Au-X1	2.401	2.402	2.403	2.399	2.406	2.273	2.275	2.276	2.272	2.275
	(2.420)	(2.421)	(2.421)	(2.418)	(2.419)	(2.292)	(2.294)	(2.295)	(2.291)	(2.293)
Au-X2	2.401	2.402	2.403	2.399	2.406	2.273	2.275	2.276	2.272	2.275
	(2.420)	(2.421)	(2.421)	(2.418)	(2.419)	(2.292)	(2.294)	(2.295)	(2.291)	(2.293)
Au-N1	2.093	2.087	2.084	2.097	2.085	2.065	2.060	2.058	2.071	2.060
	(2.073)	(2.068)	(2.068)	(2.079)	(2.071)	(2.049)	(2.04)	(2.044)	(2.055)	(2.048)
Au-N2	2.093	2.087	2.084	2.097	2.085	2.065	2.060	2.058	2.071	2.060
	(2.073)	(2.068)	(2.068)	(2.079)	(2.071)	(2.049)	(2.04)	(2.044)	(2.055)	(2.048)
N1-Au-X1	96.74	96.62	96.57	95.77	95.93	95.45	95.40	95.36	94.50	94.69
	(96.48)	(96.42)	(96.37)	(95.51)	(95.81)	(95.47)	(95.43)	(95.40)	(94.80)	(94.80)
N2-Au-X2	96.74	96.62	96.57	95.77	95.93	95.45	95.40	95.36	94.50	94.69
	(96.48)	(96.42)	(96.37)	(95.51)	(95.81)	(95.47)	(95.43)	(95.40)	(94.80)	(94.80)
X1-Au-X2	87.35	87.58	87.67	88.45	88.46	89.10	89.29	89.35	90.22	90.18
	(87.23)	(87.39)	(87.50)	(88.36)	(88.22)	(88.64)	(88.84)	(88.84)	(89.60)	(89.60)
N1-Au-N2	79.16	79.18	79.19	80.02	79.69	79.99	79.91	79.93	80.79	80.44
	(79.80)	(79.76)	(79.74)	(80.61)	(80.14)	(80.42)	(80.35)	(80.35)	(80.79)	(80.79)
C1-N1-Au-X1	180.00	180.00	180.00	180.00	179.18	180.00	180.00	180.00	180.00	179.07
	(180.00)	(180.00)	(180.00)	(180.00)	(179.68)	(180.00)	(180.00)	(180.00)	(180.00)	(179.88)
C2-N2-Au-X2	180.00	180.00	180.00	180.00	179.18	180.00	180.00	180.00	180.00	179.07
	(180.00)	(180.00)	(180.00)	(180.00)	(179.68)	(180.00)	(180.00)	(180.00)	(180.00)	(179.88)

Table S25. Eigenvalues (eV) of Kohn-Sham HOMO and LUMO calculated at DFT level (PBE0//def2-SVP/CRENBL) in the gas phase and in water solution (SCRF IEF-PCM) for the complex cations in compounds **1**–**10**.

	Gas phase		Water	
	HOMO	LUMO	HOMO	LUMO
**1**	-10.860	-7.1925	-8.0227	-4.1345
**2**	-10.645	-6.9356	-7.9811	-4.0523
**3**	-10.496	-6.7686	-7.9811	-4.0526
**4**	-10.826	-7.1310	-7.9389	-4.1495
**5**	-10.090	-6.6643	-7.4371	-4.0901
**6**	-11.437	-7.1471	-8.2753	-4.0191
**7**	-11.204	-6.8763	-8.1865	-3.9296
**8**	-11.045	-6.6943	-8.1604	-3.9280
**9**	-11.368	-7.0891	-8.0053	-4.0393
**10**	-10.167	-6.5811	-7.4654	-3.9715

Table S26. Thermochemical parameters (Hartree) for the halide exchange reaction (eq. 3 in the paper) calculated at DFT level (PBE0//def2-SVP/CRENBL) in water solution (SCRF IEF-PCM; values in parentheses) for the complex cations in compounds **1**–**10** and for the chloride and bromide anions. E_0 + ZPE = sum of electronic and zero-point energies; E_0 + ΔH = sum of electronic and thermal enthalpies; E_0+ΔG = sum of electronic and thermal free energies.

	E_0 + ZPE	E_0 + ΔH	E_0+ΔG
**1**	-5777.2423	-5777.2284	-5777.2839
**2**	-5855.6797	-5855.6627	-5855.7259
**3**	-6090.9289	-6090.9027	-6090.9850
**4**	-5853.3244	-5853.3091	-5853.3676
**5**	-6314.4064	-6314.3815	-6314.4633
**6**	-1549.9474	-1549.9332	-1549.9896
**7**	-1628.3850	-1628.3687	-1628.4289
**8**	-1863.6342	-1863.6087	-1863.6882
**9**	-1626.0289	-1626.0142	-1626.0700
**10**	-2087.1112	-2087.0869	-2087.1662
Cl^-	-460.0475	-460.0452	-460.0625
Br^-	-2573.7127	-2573.7103	-2573.7289

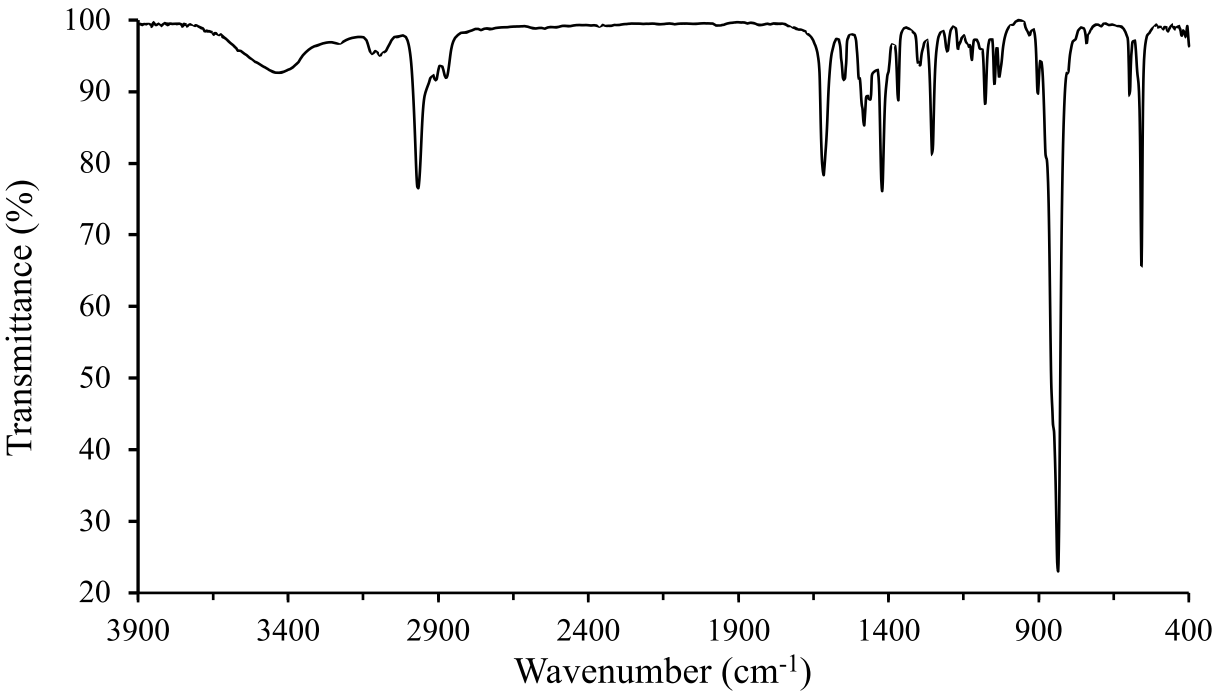

Figure S1. FT-IR spectrum of compound **3** (KBr pellet).

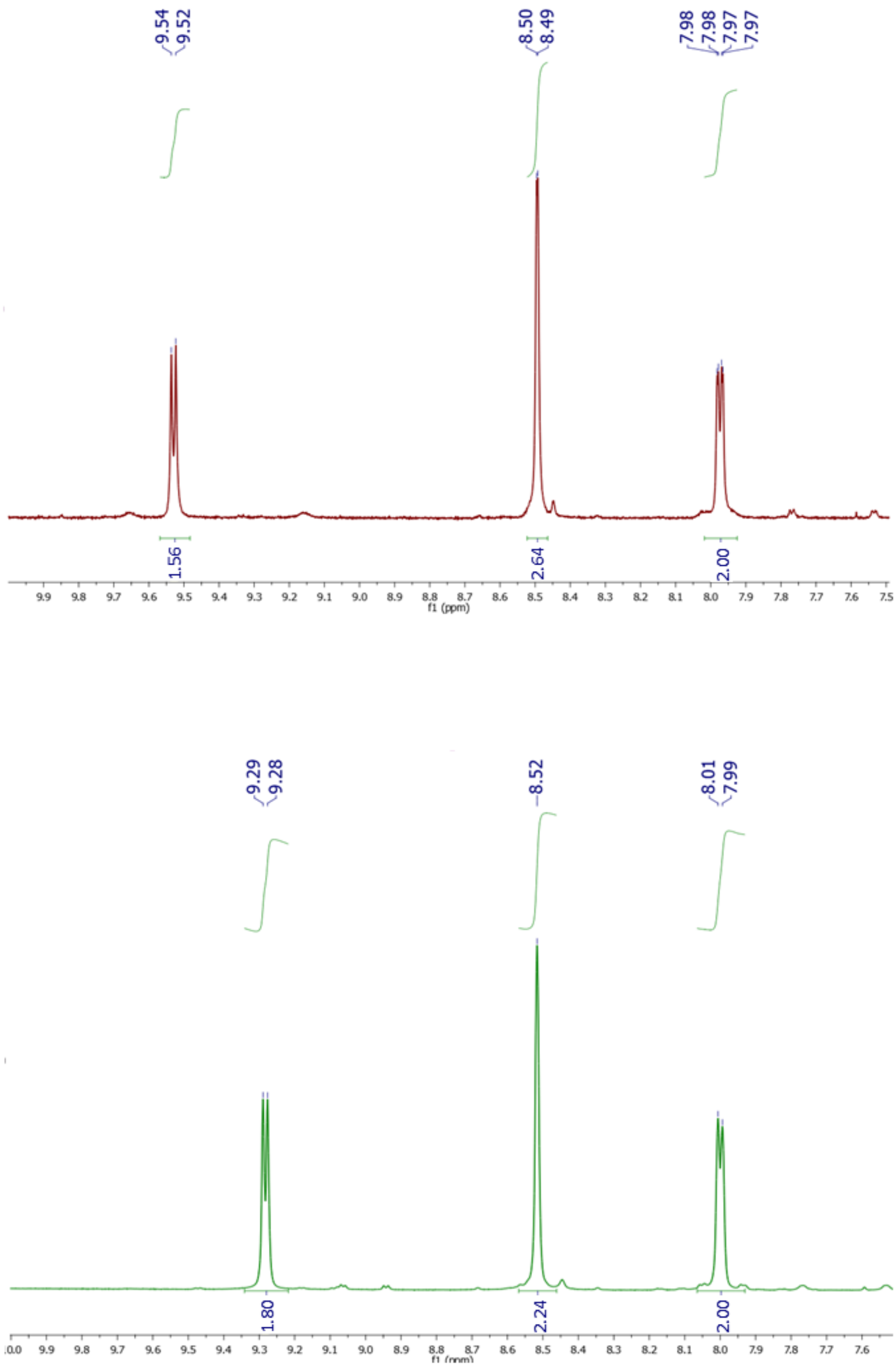

Figure S2. Detail (7.5–10 ppm) of the ^{1}H-NMR spectra (CD_3CN, 600 MHz) of compounds **3** (top) and **8** (bottom).

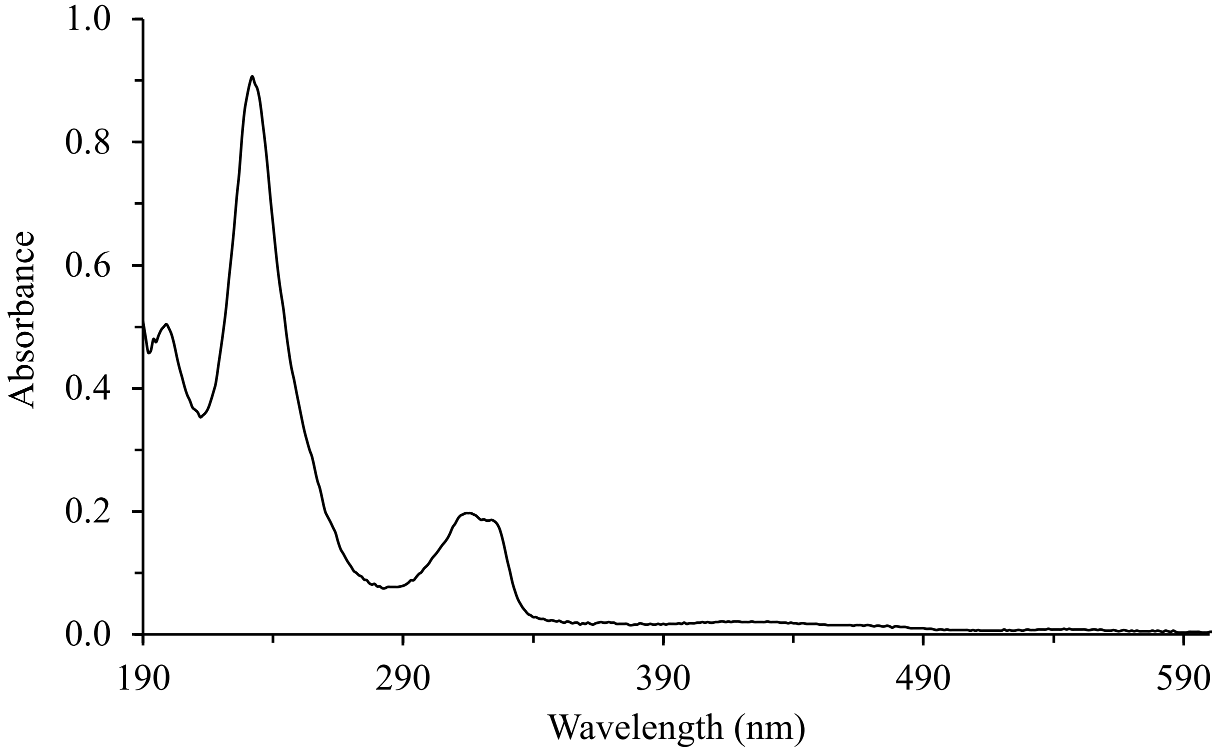

Figure S3. UV-Visible absorption spectrum (190–600 nm) recorded for compound **1** in MeCN solution ($C = 1.68\cdot10^{-5}$ M).

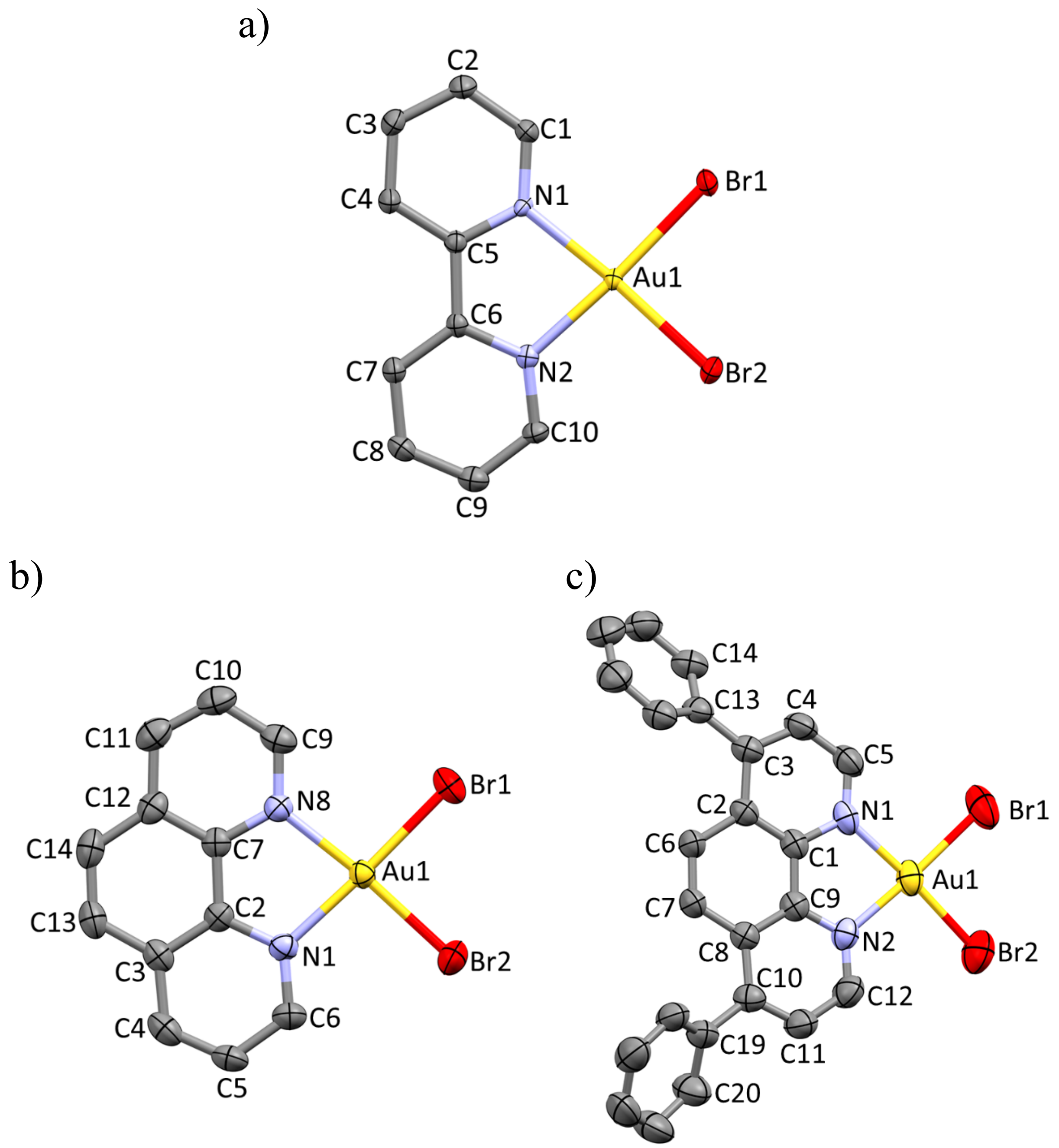

Figure S4. Drawing and atom labeling scheme for the complex cation in compounds **1** (a), **4** (b), and **5**·CH_2Cl_2 (c). Thermal ellipsoids are shown at the 60% probability level. Hydrogen atoms were omitted for clarity.

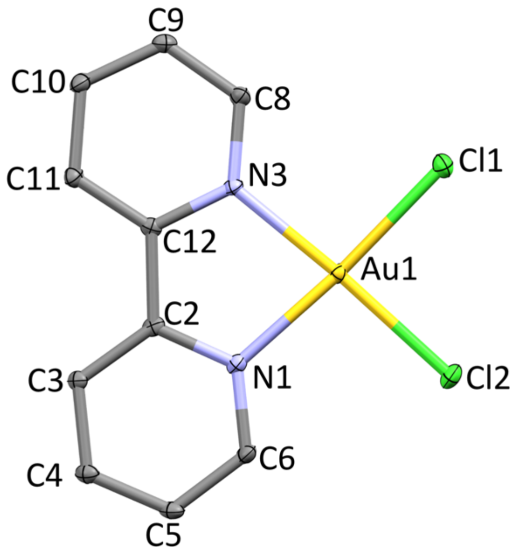

Figure S5. Drawing and atom labeling scheme for the complex cation in compounds **6** (left) and **9** (right). Thermal ellipsoids are shown at the 30% probability level. Hydrogen atoms were omitted for clarity.

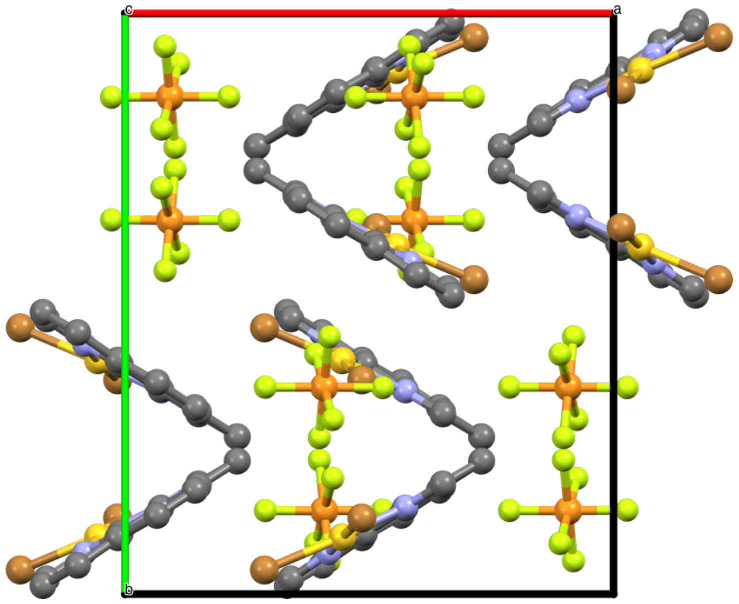

Figure S6. Portion of the packing of compound **4** viewed along the *c* axis showing the cell axis. Hydrogen atoms were omitted for clarity.

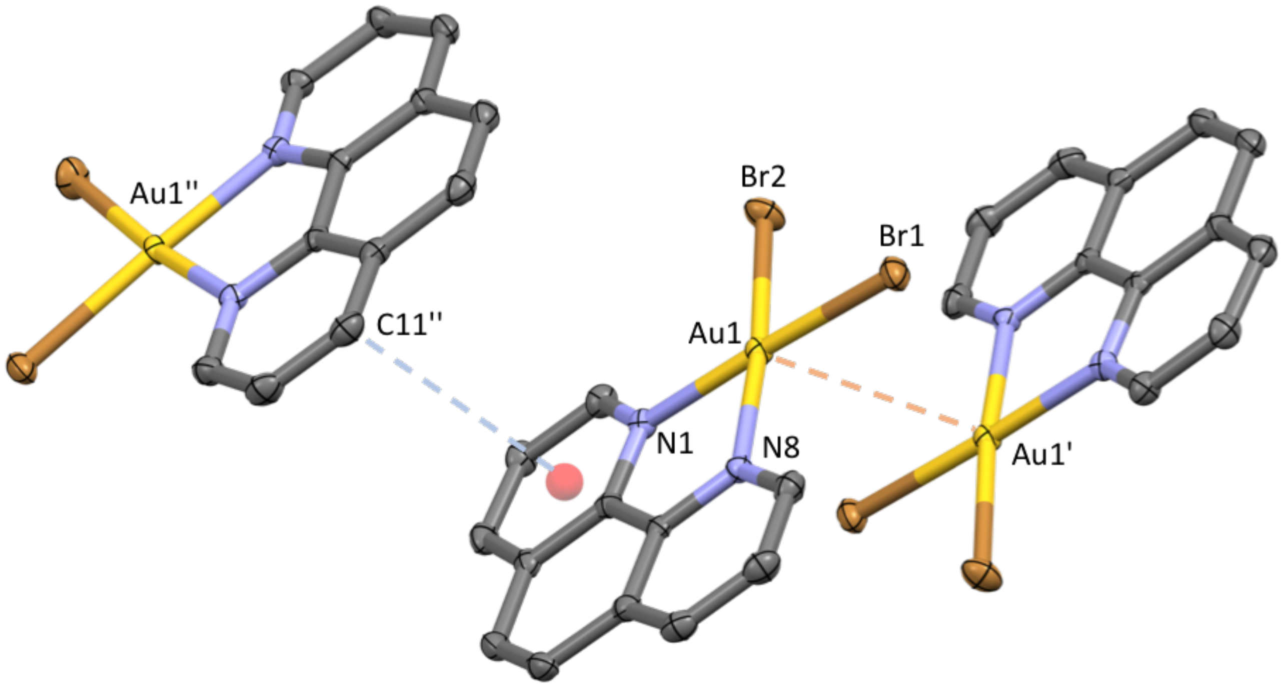

Figure S7. Portion of the packing of compound **4** showing weak aurophilic interactions Au1···Au1' (3.642 Å) and slipped π–π stacking interactions (C11···centroid, 3.654 Å). Thermal ellipsoids were shown at the 30% probability level. Hydrogen atoms were omitted for clarity. ' = 1−x, 1−y, 2−z ; '' = −1/2+x, y, 3/2−z.

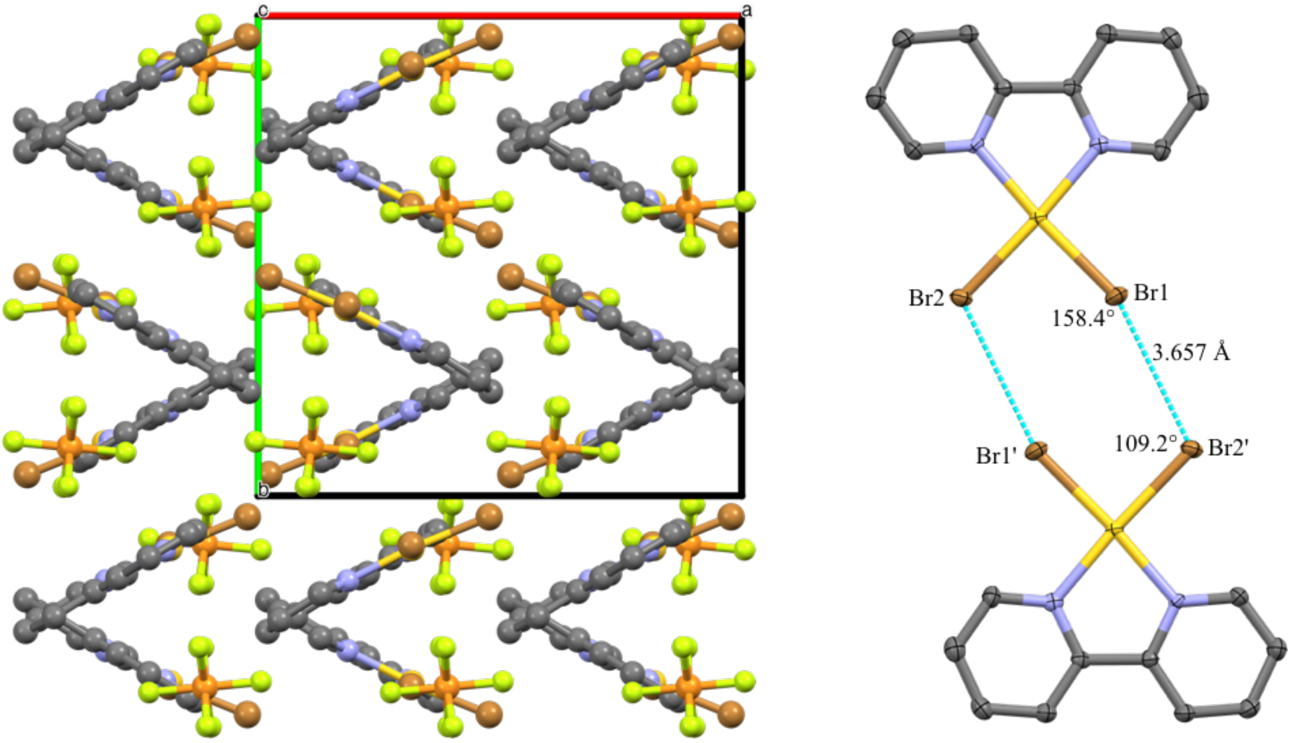

Figure S8. Portion of the packing of compound **1** viewed along the *c* axis (left) and Br···Br contacts between couples of symmetry-related complex cations (right; thermal ellipsoids shown at the 60% probability level). Hydrogen atoms were omitted for clarity. Symmetry operations: ' = 1–x, –y, 1–z.

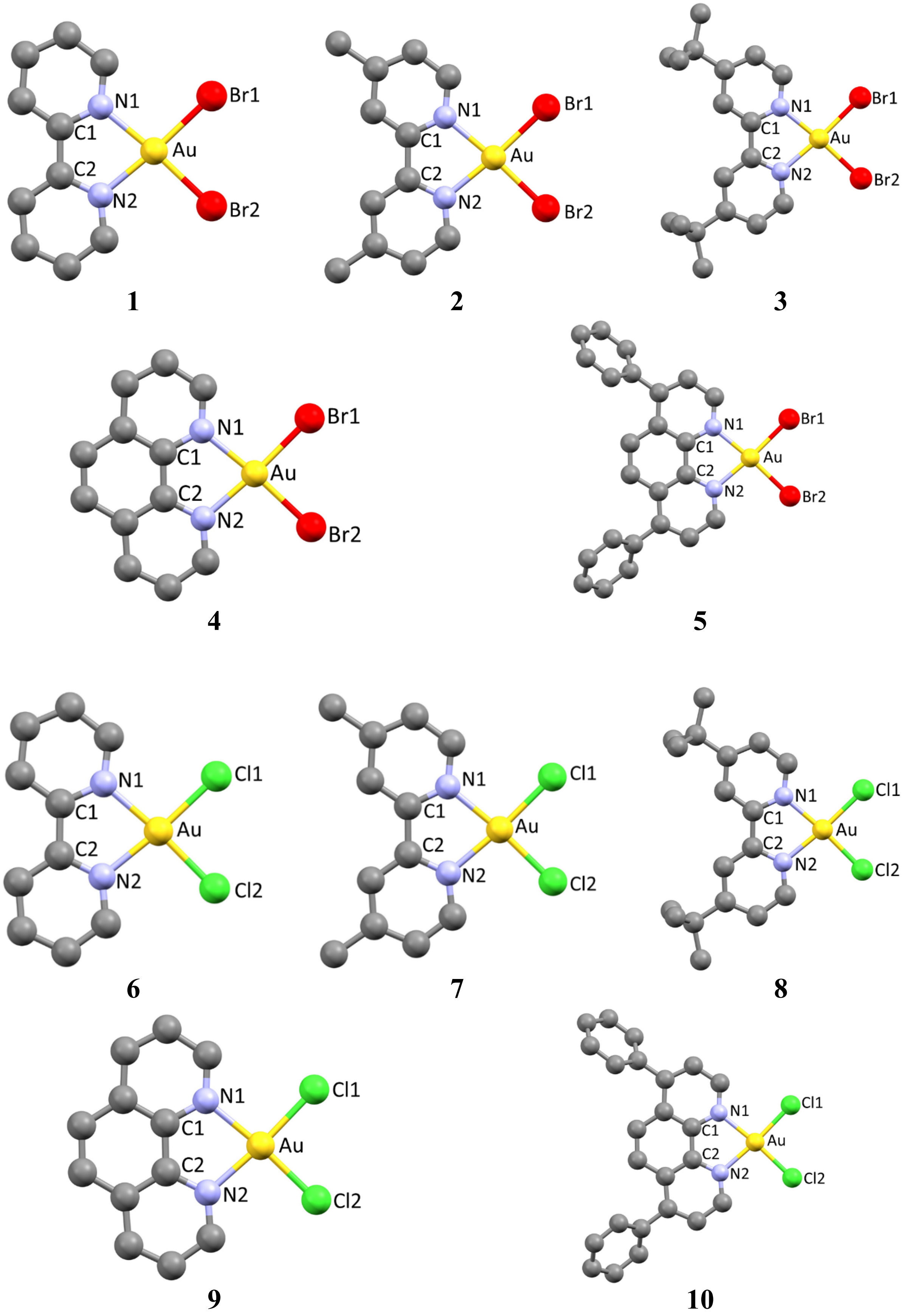

Figure S9. Molecular geometry optimized for the complex cations in compounds **1–10** at DFT level (PBE0//def2-SVP/CRENBL) in the gas phase. Hydrogen atoms were omitted for clarity.

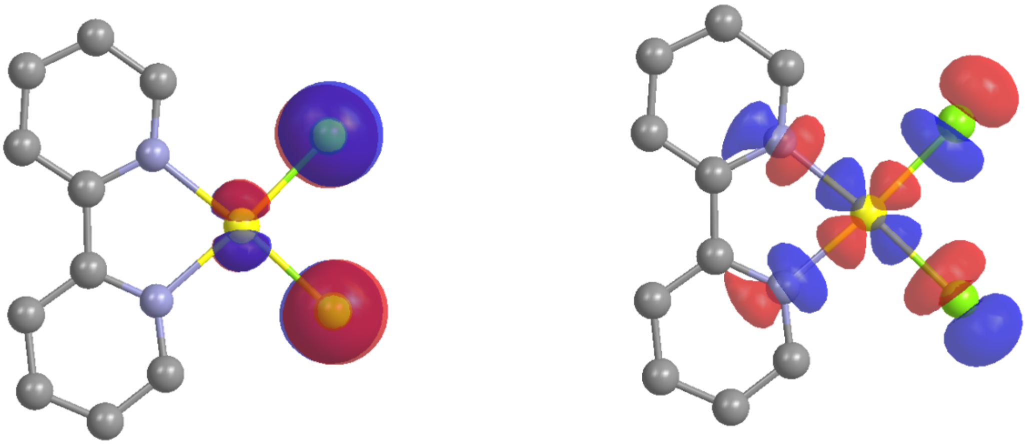

HOMO LUMO

Figure S10. Kohn-Sham frontier molecular orbitals calculated for the complex cation $[Au(bipy)Cl_2]^+$ in compound **6** at DFT level (PBE0//def2-SVP/CRENBL) in the gas phase. Hydrogen atoms were omitted for clarity; isovalue 0.05 |e|.
